# Differential disruptions in population coding along the dorsal-ventral axis of CA1 in the APP/PS1 mouse model of Aβ pathology

**DOI:** 10.1371/journal.pcbi.1012085

**Published:** 2024-05-06

**Authors:** Udaysankar Chockanathan, Krishnan Padmanabhan

**Affiliations:** 1 Department of Neuroscience, University of Rochester School of Medicine and Dentistry, Rochester, New York, United States of America; 2 Neuroscience Graduate Program, University of Rochester School of Medicine and Dentistry, Rochester, New York, United States of America; 3 Medical Scientist Training Program, University of Rochester School of Medicine and Dentistry, Rochester, New York, United States of America; 4 Ernest J. Del Monte Institute for Neuroscience, University of Rochester School of Medicine and Dentistry, Rochester, New York, United States of America; 5 Center for Visual Sciences, University of Rochester School of Medicine and Dentistry, Rochester, New York, United States of America; 6 Intellectual and Developmental Disabilities Research Center, University of Rochester School of Medicine and Dentistry, Rochester, New York, United States of America; Université Paris Descartes, Centre National de la Recherche Scientifique, FRANCE

## Abstract

Alzheimer’s Disease (AD) is characterized by a range of behavioral alterations, including memory loss and psychiatric symptoms. While there is evidence that molecular pathologies, such as amyloid beta (Aβ), contribute to AD, it remains unclear how this histopathology gives rise to such disparate behavioral deficits. One hypothesis is that Aβ exerts differential effects on neuronal circuits across brain regions, depending on the neurophysiology and connectivity of different areas. To test this, we recorded from large neuronal populations in dorsal CA1 (dCA1) and ventral CA1 (vCA1), two hippocampal areas known to be structurally and functionally diverse, in the APP/PS1 mouse model of amyloidosis. Despite similar levels of Aβ pathology, dCA1 and vCA1 showed distinct disruptions in neuronal population activity as animals navigated a virtual reality environment. In dCA1, pairwise correlations and entropy, a measure of the diversity of activity patterns, were decreased in APP/PS1 mice relative to age-matched C57BL/6 controls. However, in vCA1, APP/PS1 mice had increased pair-wise correlations and entropy as compared to age matched controls. Finally, using maximum entropy models, we connected the microscopic features of population activity (correlations) to the macroscopic features of the population code (entropy). We found that the models’ performance increased in predicting dCA1 activity, but decreased in predicting vCA1 activity, in APP/PS1 mice relative to the controls. Taken together, we found that Aβ exerts distinct effects across different hippocampal regions, suggesting that the various behavioral deficits of AD may reflect underlying heterogeneities in neuronal circuits and the different disruptions that Aβ pathology causes in those circuits.

## Author summary

Patients with Alzheimer’s disease (AD) experience an array of cognitive deficits, such as memory loss and executive dysfunction, as well as behavioral symptoms, such as agitation and anxiety. However, it is unclear how these myriad deficits arise from the molecular and cellular hallmarks of AD, such as amyloid beta (Aβ) plaques. In the current study, we sought to bridge this gap by studying how neural activity is disrupted in the context of Aβ pathology. We recorded the activity of large populations of neurons in two different brain regions, the dorsal and ventral CA1 subfields of the hippocampus, in a mouse model of Aβ pathology as animals navigated a virtual reality environment. Using statistical approaches to quantify the features of the population code, we showed that the disruptions to neuronal activity in the Aβ model were different across these two regions. These results suggest that Aβ exerts diverse effects across different brain regions that likely reflect underlying heterogeneities in the circuits of the brain.

## Introduction

Patients with Alzheimer’s disease (AD) experience an array of neurologic and psychiatric symptoms, including memory loss [1], agitation [2], and anxiety [3]. Considerable effort has been focused on identifying the molecular and cellular mechanisms that underlie these deficits. From this work, two hallmarks of the disease, amyloid β (Aβ) plaques and tau neurofibrillary tangles, have been identified [4]. Although numerous studies including those done in animal models have documented the impacts of this pathology on behavior [5–7], they leave open the critical question of how microscopic changes at the molecular and cellular level lead to the constellation of symptoms in patients with AD.

There are a number of reasons why this link remains an open area of inquiry. First, the behavioral heterogeneity seen in patients is not easily mapped to a unique cellular pathology. Second, a single pathology may affect different neuronal circuits in different ways thereby differentially impacting the behaviors that arise from these circuits. Finally, it remains challenging to relate the multi-parametric intrinsic cellular and synaptic properties of neurons (circuit structure) to the dynamics of population activity that give rise to behavior (circuit function).

One way to address these challenges is by studying changes in neuronal population activity that arise as a result of the aggregate effects of Aβ pathology on the molecular, cellular, and synaptic components of the network [8,9]. As neurophysiology arises from the molecular, cellular, and anatomical properties of the circuit, and consequently governs behavior, it serves as a bridge between these different levels of analysis. Studies of this link in an animal model require two critical elements. First, the circuit in model brain region should be structurally and functionally diverse. Second, the brain region should exhibit significant Aβ pathology. The CA1 region of the hippocampus is one area that satisfies these two conditions.

The hippocampus serves a wide array of functions, from episodic memory to affective processing [10–12]. Circuits along the longitudinal axis of the hippocampus contribute differently to these diverse functions [13], with dorsal hippocampus playing an outsized role in episodic memory and spatial cognition [14] while anxiety and social memory appear to be largely represented in ventral hippocampus [15,16]. This differentiation arises from diversity across multiple levels of organization, from gene expression [17] to neural structure and function [15,18] to afferent and efferent anatomical connectivity [19–22]. Aβ plaque accumulation and neuronal loss throughout the hippocampus has been well documented in AD [23–25].

The hippocampus is thus an anatomically and functionally heterogeneous structure that is disrupted in AD. However, most studies of hippocampal physiology, especially in AD research, have focused on the dorsal hippocampus [26]. Whether the pathophysiological effects of Aβ plaques in the ventral hippocampus are similar to those in dorsal hippocampus is largely unknown and critical for understanding how the elements of pathology at the molecular and cellular level influence behavior through the intermediary of the neural circuit.

We addressed this question by performing high-density recordings of neuronal populations in dorsal and ventral CA1 of the hippocampus in aged control and APP/PS1 mice as they ran in a virtual reality environment. Although the amount of Aβ plaques were similar in the two regions, features of population activity, such as the correlation of firing and the entropy were differentially disrupted across the longitudinal axis of the hippocampus. Our results highlight the diversity of changes in network function that arise from a single pathology, providing a framework to link molecular and cellular pathology to behavior via neurophysiology.

## Results

**Fig 1:**
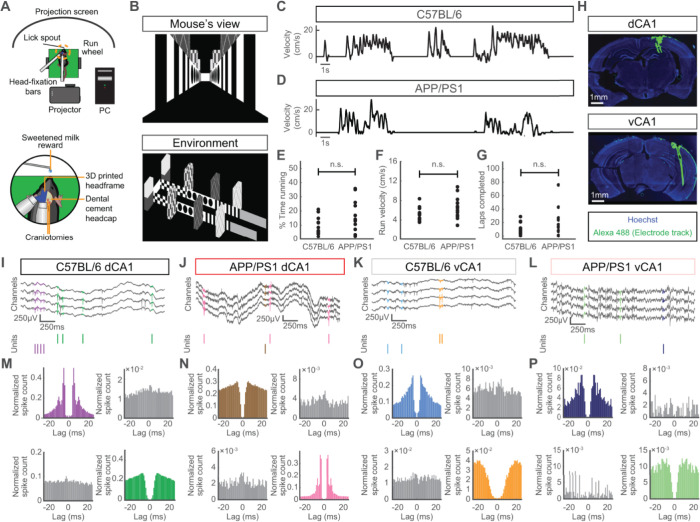
Experimental setup and spike sorting. (A) Left: schematic of head-fixed virtual reality recording rig. Right, close-up of mouse head, showing lick spout for sweetened milk reward delivery, headcap, and craniotomy sites. (B) Snapshots of virtual track, as seen from the mouse’s perspective and an overhead view. (C) Representative trace of running velocity for a C57BL/6 mouse. (D) Representative trace of running velocity for an APP/PS1 mouse. (E) There was no significant difference in the proportion of time spent running between the C57BL/6 and APP/PS1 mice (mean ± std: C57BL/6 = 9.4 ± 6.4%, APP/PS1 = 14.9 ± 10.9%, p = 0.21, two-sided Wilcoxon sign-rank test, n_C57BL/6_ = 12 recording sessions from 4 animals, n_APP/PS1_ = 15 recording sessions from 6 animals). Each point denotes a recording session. (F) There was no significant difference in the mean running velocity of the C57BL/6 and APP/PS1 mice (mean ± std: C57BL/6 = 5.2 ± 1.4 cm/s, APP/PS1 = 6.3 ± 2.3 cm/s, p = 0.16, two-sided Wilcoxon sign-rank test, n_C57BL/6_ = 12 recording sessions from 4 animals, n_APP/PS1_ = 15 recording sessions from 6 animals). Each point denotes a recording session. (G) There was no significant difference in the number of laps completed on the virtual track for the C57BL/6 and APP/PS1 mice (mean ± std: C57BL/6 = 10.3 ± 9.0, APP/PS1 = 17.4 ± 20.3, p = 0.51, two-sided Wilcoxon sign-rank test, n_C57BL/6_ = 12 recording sessions from 4 animals, n_APP/PS1_ = 15 recording sessions from 6 animals). Each point denotes a recording session. (H) Electrode arrays were coated with a fluorescent dye and targeted to either dorsal or ventral CA1. (I-L) Top: Example raw widefield electrophysiological traces in dorsal and ventral CA1 from C57BL/6 and APP/PS1 mice. In each trace, spikes from two different units are highlighted with unique colors. Bottom: Raster plot of spike times from the two units highlighted in the raw traces. (M-P) Auto-correlograms for each unit highlighted in panels M-P are shown with their respective colors. Cross-correlograms for each pair of units are shown in gray. Note the clear refractory periods in the auto-correlograms and the absence of such effects in the cross-correlogram.

To study the effects of Aβ pathology along the dorsal-ventral axis of CA1, we performed extracellular recordings in awake male C57BL/6 and APP/PS1 mice (age 14–19 months) in a virtual reality environment. Mice were head-fixed and placed on a non-motorized run-wheel. An image of a 1.9m virtual one-dimensional track was projected onto a curved screen in front of the mouse (Fig 1A and 1B), such that the movement of the wheel caused a corresponding change in the virtual track position [27,28]. When the mouse reached the end of the track, a sweetened milk reward was provided and the mouse was returned to the start of the track. There were no significant differences in running behavior, including run velocity and number of laps completed on the virtual track, between C57BL/6 and APP/PS1 mice (Fig 1C–1G). After a 7-day period in which mice were habituated to the run-wheel and virtual environment, craniotomies were performed over dorsal and ventral CA1. Following a 12-18h recovery period, the animals were placed on the run-wheel and a 128-channel electrode array was lowered into dorsal or ventral CA1 (Fig 1H). Extracellular electrophysiological recordings lasting 1–2 hours were obtained over two days, allowing the identification of spikes (action potentials) from individual neurons (Fig 1I–1L) while the animal was awake and behaving.

**Fig 2:**
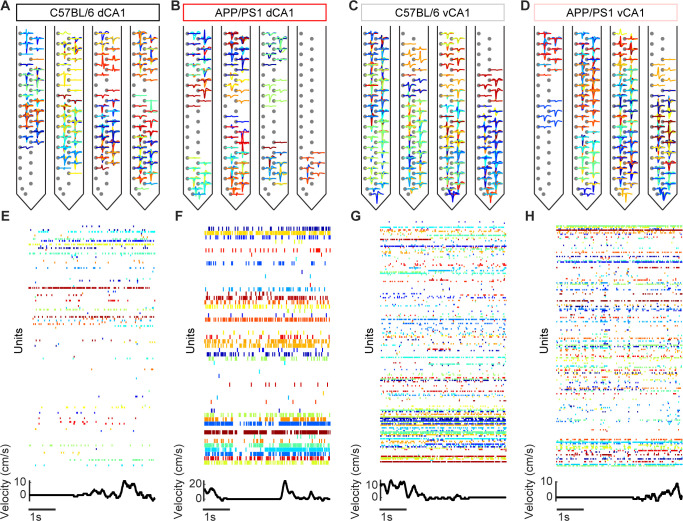
Identification of large populations of single units. (A-D) Mean waveform of all units in a representative recording session from dCA1 and vCA1 in C57BL/6 and APP/PS1 animals. For each unit, waveforms are shown on the channel with the largest amplitude spike as well as up to four neighboring channels. (E-H) Top: Sample of population activity from each of the four groups, where the color of a given row on the raster plot refers to the mean waveform of that unit in panels a-d. Bottom: Simultaneous running velocity of the mouse.

From the band passed raw signals, we performed spike sorting across multiple neighboring channels using Kilosort 2 [29] and the resulting putative units (neurons) were manually curated using Phy 2 [30]. Only units with clear refractory periods in their auto-correlograms (Fig 1M–1P) and characteristic mean waveforms (Fig 2A–2D) were included in subsequent analyses. Large populations of simultaneously recorded neurons were identified in dCA1 and vCA1 in both C57BL/6 and APP/PS1 mice (Fig 2E–2H). From each dCA1 recording session, 33–133 units were identified in C57BL/6 mice and 26–44 units were identified in APP/PS1 mice. From the vCA1 recording sessions, an average of 61–178 units were identified in C57BL/6 mice and 29–174 units were identified in APP/PS1 mice. Additionally, units were classified as excitatory or inhibitory based on the trough-to-peak time of their mean waveform (S1 Fig) [31,32]. In dCA1, excitatory units comprised 73% of units in C57BL/6 mice and 60% of units in APP/PS1 mice, while in vCA1, excitatory units comprised 61% of units in C57BL/6 mice and 59% of units in APP/PS1 mice. There was no significant difference in the proportion of excitatory or inhibitory units between C57BL/6 and APP/PS1 mice in either dCA1 or vCA1.

**Fig 3:**
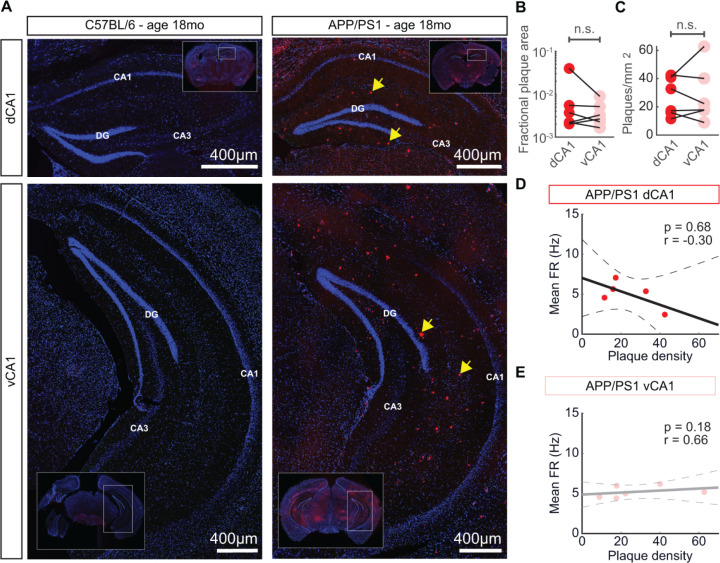
No significant differences in Aβ plaque burden between dCA1 and vCA1 in APP/PS1 mice. (A) Representative histological sections with from C57BL/6 and APP/PS1 mice with Congo Red staining for Aβ plaques (red) and Hoechst staining for cell nuclei (blue). Aβ plaques are present in both dorsal and ventral CA1 in APP/PS1 animals, but not in C57BL/6 animals. Yellow arrows indicate individual Aβ plaques. (B) There were no significant differences in fractional Aβ plaque area between dorsal and ventral CA1 in APP/PS1 mice (mean ± std: dCA1 = 9.2×10^−3^ ± 1.5×10^−2^, vCA1 = 4.0×10^−3^ ± 2.7×10^−3^, p > 0.99, two-sided Wilcoxon sign-rank test, n = 6 animals). Each point denotes a single animal. (C) There were no significant differences in plaque density between dorsal and ventral CA1 in APP/PS1 mice (mean ± std: dCA1 = 26.8 ± 13.6, vCA1 = 28.2 ± 19.8, p > 0.99, two-sided Wilcoxon sign-rank test, n = 6 animals). Each point denotes a single animal. (D) There was no significant correlation between the mean firing rate of dCA1 neurons and the dCA1 Aβ plaque density (r = -0.30, p = 0.68, Spearman rank correlation coefficient, n = 5 animals). Each point denotes a single animal. The black solid line denotes the least-squares regression and the black dashed lines denote the boundaries of the 95% confidence interval of the regression. (E) There was no significant correlation between the mean firing rate of vCA1 neurons and the vCA1 Aβ plaque density (r = 0.66, p = 0.18, Spearman rank correlation coefficient, n = 6 animals). Each point denotes a single animal. The grey solid line denotes the least squares regression and the grey dashed lines denote the boundaries of the 95% confidence interval of the regression.

After recordings were completed, mice were sacrificed and the brain was removed and sectioned to confirm the placement of the probe in either dCA1 or vCA1 and stained for Aβ plaques with Congo Red. Aβ plaques were identified throughout the dorsoventral axis of the hippocampus and cortex in APP/PS1 mice, but not in C57BL/6 animals (Fig 3A). Sections were digitized using a slide scanner and plaques were manually circumscribed. We found no significant difference in relative Aβ plaque area (Fig 3B) or density of Aβ plaques (Fig 3C) between dCA1 and vCA1. Furthermore, we found no association between the Aβ plaque burden and the mean firing rate of neurons in either dCA1 or vCA1 (Figs 3D, 3E and S2). This was consistent with earlier studies demonstrating that the effects of Aβ pathology on neuronal activity could be heterogeneous in cortical populations [33].

**Fig 4:**
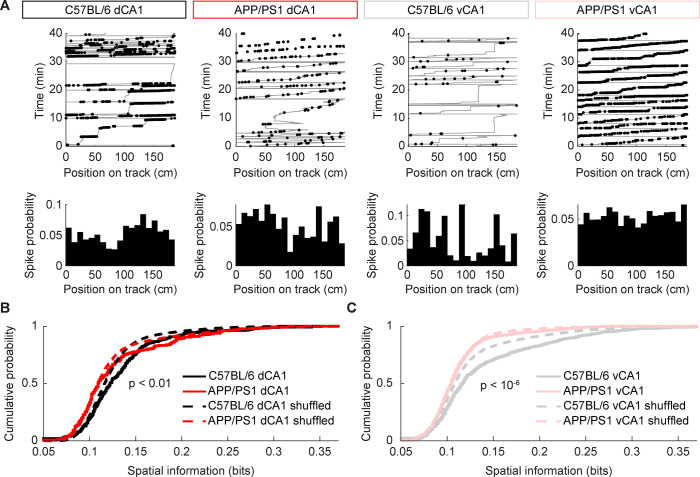
Spatial information is significantly decreased in dCA1 and vCA1 in APP/PS1 mice. (A) Spiking activity of representative neurons (black points) overlaid with animal trajectories on the virtual track (gray lines). Histograms show the mean firing rate for the corresponding neuron along each segment of the virtual track. (B) In dCA1, spatial information was decreased in APP/PS1 mice relative to C57BL/6 controls (mean ± std: C57BL/6 = 0.132 ± 0.048, APP/PS1 = 0.128 ± 0.051, p < 0.005, two-sided Wilcoxon rank-sum test, n_C57BL/6_ = 305 units from 5 recording sessions, n_APP/PS1_ = 180 units from 4 recording sessions). The spatial information in dCA1 was significantly larger than circularly shuffled spike trains with similar mean firing rates for C57BL/6 mice (mean ± std: empirical = 0.132 ± 0.048, shuffled = 0.124 ± 0.035, p < 0.001, two-sided Wilcoxon rank-sum test, n_empirical_ = 305 units from 5 recording sessions, n_shuffled_ = 30500 simulated units from 5 recording sessions), but not for APP/PS1 mice (mean ± std: empirical = 0.128 ± 0.051, shuffled = 0.123 ± .047, p = 0.39, two-sided Wilcoxon rank-sum test, n_empirical_ = 180 units from 4 recording sessions, n_shuffled_ = 18000 simulated units from 4 recording sessions). (C) In vCA1, spatial information was decreased in APP/PS1 mice relative to C57BL/6 controls (mean ± std: C57BL/6 = 0.138 ± 0.065, APP/PS1 = 0.112 ± 0.035, p < 10^−6^, two-sided Wilcoxon rank-sum test, n_C57BL/6_ = 689 units from 6 recording sessions, n_APP/PS1_ = 651 units from 8 recording sessions). The spatial information in vCA1 was significantly larger than circularly shuffled spike trains with similar mean firing rates for C57BL/6 mice (mean ± std: empirical = 0.138 ± 0.065, shuffled = 0.123 ± .049, p < 10^−6^, two-sided Wilcoxon rank-sum test, n_empirical_ = 689 neurons from 6 recording sessions, n_shuffled_ = 68900 simulated neurons from 6 recording sessions), but not for APP/PS1 mice (mean ± std: empirical = 0.112 ± 0.035, shuffled = 0.110 ± 0.031, p < 0.89, two-sided Wilcoxon rank-sum test, n_empirical_ = 651 neurons from 8 recording sessions, n_shuffled_ = 65100 simulated neurons from 8 recording sessions).

As the mean firing rate may have been insufficient to capture the effects of Aβ on neuronal activity, we chose to approach the problem from the perspective of information coding. The neurons of the hippocampus have been shown to encode information about social interactions, anxiety, and spatial position [16,34,35]. We focused on spatial representations given the well documented deficit in spatial cognition in patients with AD [36] as well as in mouse models [37]. In our experiments, we did not find evidence of classic “place” cells, neurons that preferentially activate when an animal visits a particular region of its environment (Figs 4A and S3). Importantly, we found no neurons in either dCA1 or vCA1 that repeatedly activated on the same portion of the virtual track across multiple laps (Fig 4A). We quantified this using a spatial stability score [38,39] and found that the stability of spatial tuning was decreased in APP/PS1 mice across both hippocampal subfields. However, the majority of neurons in both groups had a stability score of near zero, indicating that the spatial tunings were different between the first and second halves of a given recording session (S4 Fig). This may have been partially due to the advanced age of the animals, which has been associated with degraded place cell tunings relative to younger animals [26]. Moreover, in head-fixed setups, animals do not receive vestibular inputs or airflow sensation over the vibrissae; they must rely on vision and proprioceptive inputs. Taken together with prior observations of age-related impairments in mouse visual acuity [40,41] and proprioception [42], these restrictions may substantially impair the spatial cognition of aged animals in virtual environments and could explain the absence of clear place fields in our experiment.

Prior studies of hippocampal activity in both real-world and virtual environments have illustrated that neurons without clear place fields can nevertheless influence spatial coding by shaping, for example, the trial-to-trial variability of place cell sequences [43,44]. Indeed, we noticed that the statistical properties of the neuronal responses across regions and between control and APP/PS1 animals appeared different. To take a model agnostic perspective, we calculated the spatial information, which quantifies the extent to which a neuron’s firing rate decreases the uncertainty in the animal’s position, allowing us to identify coding properties of individual cells regardless of if they had a classically defined place field. We found that the spatial information of neurons in APP/PS1 mice was lower than that in C57BL/6 mice in dCA1 (Fig 4B), consistent with results from prior studies in freely moving mice [26,45]. In vCA1 neurons, we also found that APP/PS1 mice had lower spatial information than controls (Fig 4C). For C57BL/6 neurons in both regions, the average spatial information was significantly larger than would be expected from circularly shuffled spike trains. However, the average spatial information in APP/PS1 neurons was not significantly different from chance in either dCA1 or vCA1, which lends further evidence to suggest that spatial information is degraded in the context of Aβ pathology. These results also held true even when considering only excitatory neurons (S5 Fig). Moreover, as the mean and variance of neuronal firing rates in both dCA1 and vCA1 were similar for C57BL/6 and APP/PS1 mice (S6 and S7 Figs), these findings are likely not a simple result of variations in the average activity between the two strains. Rather, they demonstrate a degradation in the hippocampal spatial information content of dCA1 and vCA1 neurons in the context of Aβ pathology.

**Fig 5:**
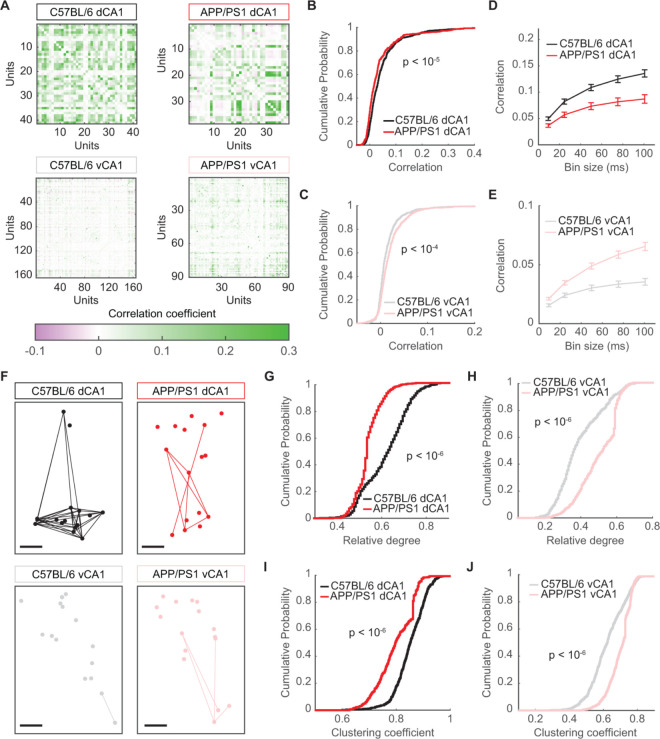
Pairwise correlations in APP/PS1 mice are decreased in dCA1, but increased in vCA1. (A) Matrices of correlation coefficients for all neurons in representative recording sessions. (B) In dCA1, mean correlations were lower in APP/PS1 mice than in C57BL/6 mice (mean ± std: C57BL/6 = 0.047 ± 0.066, APP/PS1 = 0.034 ± 0.063, p < 10^−5^, two-sided Wilcoxon rank-sum test, n_C57BL/6_ = 500 unit pairs from 5 recording sessions, n_APP/PS1_ = 400 unit pairs from 4 recording sessions). (C) In vCA1, mean correlations were higher in APP/PS1 mice than in C57BL/6 mice (mean ± std: C57BL/6 = 0.014 ± 0.02, APP/PS1 = 0.021 ± 0.033, p < 10^−4^, two-sided Wilcoxon rank-sum test, n_C57BL/6_ = 600 unit pairs from 6 recording sessions, n_APP/PS1_ = 800 unit pairs from 8 recording sessions). (D) In dCA1, APP/PS1 mice had significantly lower correlations than C57BL/6 mice when spiking activity was binned at 5ms, 25ms, 50ms, 75ms, and 100ms (for all bin sizes, p < 10^−5^, two-sided Wilcoxon rank-sum test, n_C57BL/6_ = 500 unit pairs from 5 recording sessions, n_APP/PS1_ = 400 unit pairs from 4 recording sessions). Error bars show the standard error of the mean. (E) In vCA1, APP/PS1 mice had significantly higher correlations than C57BL/6 mice when spiking activity was binned at 5ms, 25ms, 50ms, 75ms, and 100ms (for all bin sizes, p < 10^−6^, two-sided Wilcoxon rank-sum test, n_C57BL/6_ = 600 unit pairs from 6 recording sessions, n_APP/PS1_ = 800 unit pairs from 8 recording sessions). Error bars show the standard error of the mean. (F) Graph visualizations of 16-unit neuronal populations. Circles denote neurons and lines denote correlations that exceeded a threshold of 0.06. The spatial position of each neuron corresponds to its approximate relative physical location. Scale bars denote 100μm. (G) In dCA1 graphs, the mean degree was smaller in APP/PS1 mice than in C57BL/6 mice (mean ± std: C57BL/6 = 0.66 ± 0.10, APP/PS1 = 0.54 ± 0.08, p < 10^−6^, two-sided Wilcoxon rank-sum test, n_C57BL/6_ = 500 samples from 5 recording sessions, n_APP/PS1_ = 400 samples from 4 recording sessions). (H) In vCA1 graphs, the mean degree was larger in APP/PS1 mice than in C57BL/6 mice (mean ± std: C57BL/6 = 0.48 ± 0.15, APP/PS1 = 0.57 ± 0.13, p < 10^−6^, two-sided Wilcoxon rank-sum test, n_C57BL/6_ = 600 samples from 6 recording sessions, n_APP/PS1_ = 800 samples from 8 recording sessions). (I) In dCA1 graphs, the clustering coefficient was smaller in APP/PS1 mice than in C57BL/6 mice (mean ± std: C57BL/6 = 0.85 ± 0.06, APP/PS1 = 0.81 ± 0.07, p < 10^−6^, two-sided Wilcoxon rank-sum test, n_C57BL/6_ = 500 samples from 5 recording sessions, n_APP/PS1_ = 400 samples from 4 recording sessions). (J) In vCA1 graphs, the clustering coefficient was larger in APP/PS1 mice than in C57BL/6 mice (mean ± std: C57BL/6 = 0.69 ± 0.13, APP/PS1 = 0.76 ± 0.09, p < 10^−6^, two-sided Wilcoxon rank-sum test, n_C57BL/6_ = 600 samples from 6 recording sessions, n_APP/PS1_ = 800 samples from 8 recording sessions).

The fact that disruptions in spatial information occurred in the absence of clear place cells speaks to the distributed nature of hippocampal representations. Several recent studies have provided evidence that spatial coding in the hippocampus is not done merely by individual cells, but rather involves the correlated activity of neuronal populations (including place and non-place cells) [43,44]. Moreover, disruptions in neuronal correlations have been associated with an array of neurologic disorders [46–48]. We thus hypothesized that one consequence of Aβ pathology would be an alteration in the functional connectivity between neurons. We first calculated the activity correlations between pairs of neurons (Fig 5A) and found that pairwise correlations in dCA1 were decreased in APP/PS1 mice, relative to C57BL/6 mice (Fig 5B), consistent with earlier studies [49]. Interestingly, however, in vCA1, correlations in the APP/PS1 animals were larger than those in C57BL/6 animals (Fig 5C), suggesting a differential effect of Aβ pathology on the functional organization of neuronal connections across the dorsoventral hippocampal axis. These results were obtained using a temporal bin size of 10ms. However, the findings held true across a range of bin sizes up to 100ms (Fig 5D and 5E).

As with metrics like firing rate, mean changes in pairwise correlations and functional connectivity represent the complex architecture of networks as a single value, which can often obscure critical aspects of network topology necessary to understand function. Inspired by studies from functional magnetic resonance imaging (fMRI) [50–52] and previous work using graph theoretical approaches to study functional interactions across neuronal populations in CA1 [28], we built on our correlation result by studying the topology of the functional connectivity we observed in our recordings. To do this, we constructed network graphs of the populations [53], in which nodes denoted neurons and edges denoted a pairwise correlation greater than 0.06 (Fig 5F). For each recording session, we took 100 16-unit subsamples of the overall population and calculated the average degree, or number of incident edges on each node, of the network. We found that in dCA1 networks, the average degree in APP/PS1 mice was smaller than that in C57BL/6 mice (Fig 5G). By contrast, in vCA1, the network degree in APP/PS1 mice was larger than that in C57BL/6 mice (Fig 5H). We found a similar result when calculating the clustering coefficient, which quantifies the extent to which the nodes of a network form densely connected groups. Dorsal CA1 networks in APP/PS1 animals were less clustered than those in C57BL/6 animals (Fig 5I), while vCA1 networks in APP/PS1 animals were more clustered than those in C57BL/6 animals (Fig 5J). These correlation and network structure results held true even after excluding interneurons and considering only excitatory neurons (S8 Fig). Taken together, these findings demonstrate that the nature of network disruption in APP/PS1 mice was contingent on the brain region being studied, with dCA1 populations becoming less synchronous and vCA1 populations becoming more synchronous.

While pairwise correlations play a critical role in shaping the global patterns of activity [44,54], neuronal populations or ensembles exhibit complex interactions that extend beyond just pairs of neurons [55]. Previous studies have shown that studying ensemble activity can be critical for understanding sensory perception [56–59], social cognition [60,61], and spatial navigation [43,44]. Additionally, two recent studies showed that changes in the statistical distributions of ensembles can serve as a marker for neuropsychiatric diseases [46,49]. Ensemble patterns of activity across populations of neurons thus represent essential features of network function. We therefore sought to understand whether the properties of ensembles differed between C57BL/6 and APP/PS1 mice and how these disruptions varied across dorsal and ventral CA1.

**Fig 6:**
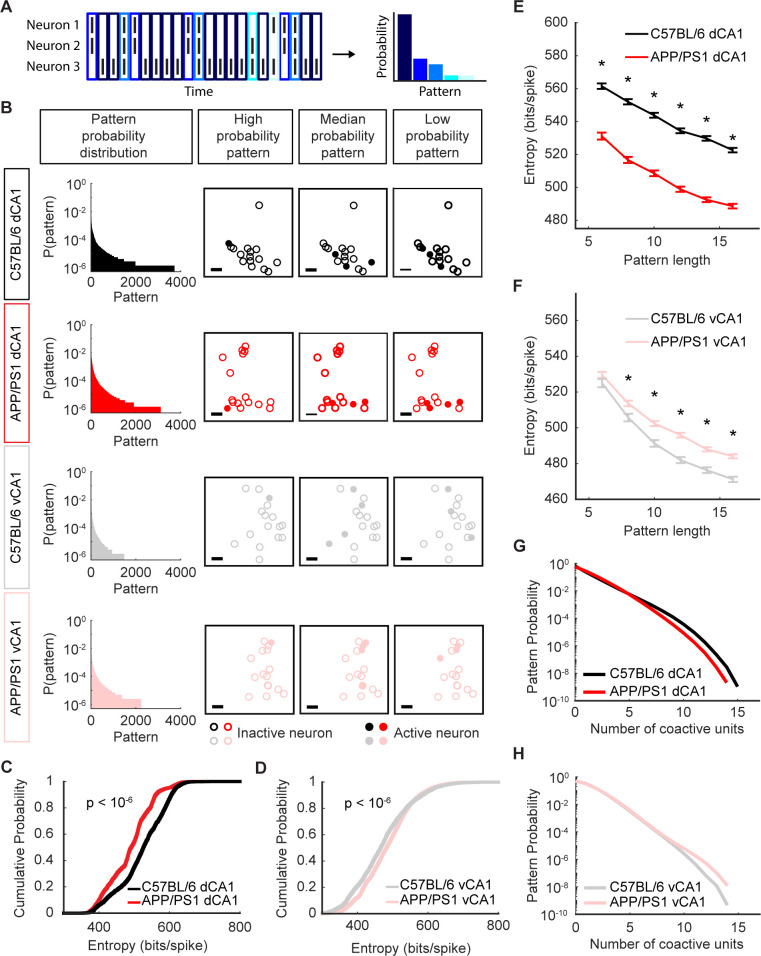
Population entropy in APP/PS1 mice is decreased in dCA1, but increased in vCA1. (A) Schematic of pattern identification and quantification using a 3-neuron cartoon population. A pattern is the combination of active and inactive neurons within a 10ms non-overlapping time window and can be conceptualized as a vertical column of the population spiking raster. In this 3-neuron population, there are 2^3^ possible patterns, though only 5 appear during this sample. Each pattern is assigned a unique shade of blue in the raster and in the probability distribution. (B) Left: representative examples of 16-neuron pattern probability distributions from dorsal and ventral CA1 in C57BL/6 and APP/PS1 mice. Right: visualizations of high, median, and low probability example patterns drawn from the corresponding probability distribution on the left. Open circles indicate inactive neurons and filled circles denote active neurons. The spatial position of each circle corresponds to its approximate relative physical position (jitter added to better visualize overlapping neurons). Scale bars denote 100μm. (C) In dCA1 populations, the entropy was lower in APP/PS1 mice than in C57BL/6 mice (mean ± std: C57BL/6 = 522 ± 68 bits/spike, APP/PS1 = 489 ± 62 bits/spike, p < 10^−6^, two-sided Wilcoxon rank-sum test, n_C57BL/6_ = 2500 samples from 5 recording sessions, n_APP/PS1_ = 2000 samples from 4 recording sessions). These data were generated using 16-unit subpopulations. (D) In vCA1 populations, the entropy was higher in APP/PS1 mice than in C57BL/6 mice (mean ± std: C57BL/6 = 471 ± 82 bits/spike, APP/PS1 = 484 ± 72 bits/spike, p < 10^−6^, two-sided Wilcoxon rank-sum test, n_C57BL/6_ = 3000 samples from 6 recording sessions, n_APP/PS1_ = 4000 samples from 8 recording sessions). These data were generated using 16-unit subpopulations. (E) In dCA1, APP/PS1 mice had a significantly lower entropy with subpopulation sizes of 6, 8, 10, 12, 14, and 16 (asterisks denote p < 10^−6^, two-sided Wilcoxon rank-sum test, n_C57BL/6_ = 2500 subsamples from 5 recording sessions, n_APP/PS1_ = 2000 subsamples from 4 recording sessions). Error bars show the standard error of the mean. (F) In vCA1, APP/PS1 mice had a significantly higher entropy with subpopulation sizes of 8, 10, 12, 14, and 16 (asterisks denote p < 10^−3^, two-sided Wilcoxon rank-sum test, n_C57BL/6_ = 3000 subsamples from 6 recording sessions, n_APP/PS1_ = 4000 subsamples from 8 recording sessions). Error bars show the standard error of the mean. (G) Probability distributions for dCA1 patterns grouped by number of coactive units. Pattern probabilities were averaged across 2500 subsamples from 5 recording sessions in C57BL/6 mice and across 2000 subsamples from 4 recording sessions in APP/PS1 mice. These data were generated using 16-unit subpopulations. (H) Probability distributions for vCA1 patterns grouped by number of coactive units. Pattern probabilities were averaged across 3000 samples from 6 recording sessions in C57BL/6 mice and across 4000 samples from 8 recording sessions in APP/PS1 mice. These data were generated using 16-unit subpopulations.

One approach to do this is to simply quantify the distribution of patterns that are observed in the population. If we define a pattern as a combination of active neurons (represented as 1) and inactive neurons (represented as a 0) (Fig 6A), then in a 16-neuron population, there would be 2^16^ possible patterns. Each of these patterns can be thought of as a state of the network, and the probability distribution of the patterns thus reflects organization of the neuronal population. Some patterns will occur more frequently than others; for example, given the sparsity of neuronal activity, periods of silence, when all of the neurons are inactive, will occur more often than periods with 12–14 coactive neurons (Fig 6B). When we compared the probability distributions of patterns of activity between C57BL/6 and APP/PS1 mice using 16-neuron subpopulations, interesting themes emerged. In dCA1, the distributions were narrower, indicating a less diverse set of patterns in APP/PS1 mice as compared to C57BL/6 mice. By contrast, in vCA1, the pattern distributions were broader in APP/PS1 mice as compared to those seen in C57BL/6 mice (Fig 6B). One way to quantify these distributions is to calculate the entropy (see Materials and Methods). We found that patterns of activity in dCA1 were much more diverse in C57BL/6 animals, leading to a higher entropy as compared to the APP/PS1 animals (Fig 6C). Surprisingly, in vCA1, the entropy of the pattern distributions was higher in APP/PS1 mice as compared to C57BL/6 mice, indicating a more diverse set of patterns (Fig 6D). These differences in entropy held true across a range of subpopulation sizes (Fig 6E and 6F). To ensure that the resultant differences were not merely a consequence of differences in mean neuronal activity, we verified that these results held true even after matching the mean firing rates of the subpopulations in the C57BL/6 and APP/PS1 groups (S9 Fig). These results therefore reflected underlying differences in the number of different states or patterns that populations of neurons visited as the population activity evolved (Fig 6G and 6H). These data suggest that the diversity of patterns observed reflected an underlying principle of network organization across the hippocampus and that the organization is differentially affected in dCA1 versus vCA1.

**Fig 7:**
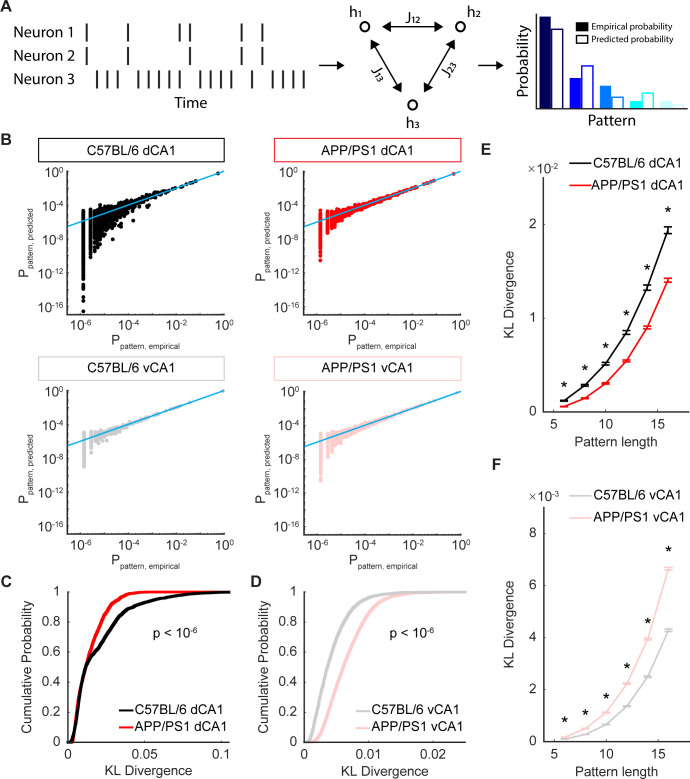
Prediction of pattern probabilities by pairwise interactions in APP/PS1 mice is improved in dCA1, but worsened in vCA1. (A) Schematic of maximum entropy model. From the population spike rasters, two sets of terms were fitted: a set of activity terms *h*_*i*_ and a set of pairwise interaction terms *J*_*ij*_. From these terms, a synthetic pattern probability distribution was generated in which the mean activity of each neuron and the coactivity of every pair of neurons was identical to that of the empirical distribution but was otherwise unstructured. The predicted and empirical pattern probabilities were then compared. (B) Representative examples of maximum entropy model predicted probabilities and empirical probabilities, generated using 16-unit subpopulations. Each point denotes a single pattern and the grey line denotes unity. (C) In dCA1 populations, the KLD was lower in APP/PS1 mice than in C57BL/6 mice (mean ± std: C57BL/6 = 1.9×10^−2^ ± 1.8×10^−2^, APP/PS1 = 1.4×10^−2^ ± 9.3×10^−3^ bits/spike, p < 10^−6^, two-sided Wilcoxon rank-sum test, n_C57BL/6_ = 2500 samples from 5 recording sessions, n_APP/PS1_ = 2000 samples from 4 recording sessions). These data were generated using 16-unit subpopulations. (D) In vCA1 populations, the entropy was higher in APP/PS1 mice than in C57BL/6 mice (mean ± std: C57BL/6 = 4.3×10^−3^ ± 2.7×10^−3^, APP/PS1 = 6.6×10^−3^ ± 3.3×10^−3^, p < 10^−4^, two-sided Wilcoxon rank-sum test, n_C57BL/6_ = 3000 samples from 6 recording sessions, n_APP/PS1_ = 4000 samples from 8 recording sessions). These data were generated using 16-unit subpopulations. (E) In dCA1, APP/PS1 mice had a significantly lower KLD with subpopulation sizes of 6, 8, 10, 12, 14, and 16 (asterisks denote p < 10^−4^, two-sided Wilcoxon rank-sum test, n_C57BL/6_ = 2500 subsamples from 5 recording sessions, n_APP/PS1_ = 2000 subsamples from 4 recording sessions). Error bars show the standard error of the mean. (F) In vCA1, APP/PS1 mice had a significantly higher KLD with subpopulation sizes of 6, 8, 10, 12, 14, and 16 (asterisks denote p < 10^−6^, two-sided Wilcoxon rank-sum test, n_C57BL/6_ = 3000 subsamples from 6 recording sessions, n_APP/PS1_ = 4000 subsamples from 8 recording sessions). Error bars show the standard error of the mean.

Calculations of entropy are an accounting of the macroscopic states or patterns generated by ensembles of neurons, while the time varying correlations shown in Fig 5 provide a microscopic detailing of the features of activity that might be constraining and shaping those states. We wanted to see if the pairwise interactions that reflect neuronal activity at the microscopic scale could be used to predict the macroscopic distributions of activity across the populations. To do this, we turned to a class of models called maximum entropy models, which aim to predict the distribution of patterns that occur across neural population while assuming as little as possible about the interactions governing that population [44,54,55,62]. In the second order models used here, the goal is to predict the probability distribution of patterns observed by considering only the activity (firing rate) of each neuron and the pairwise correlation between neurons. The model uses two sets of parameters: [1] a *h*_*i*_ term for each neuron that describes its mean activity level and [2] a *J*_*ij*_ term for every pair of neurons that describe their coactivity. Using these terms, we generated a synthetic probability distributions for all possible patterns and then compared the probabilities from the model to those measured in the data, again using 16-neuron subpopulations (Fig 7A). We visualized the synthetic and empirical probabilities using scatter plots, in which each point corresponded to a single pattern (Fig 7B), and we quantified the difference between the two probability distributions (data vs. model) using the Kulback-Leibler divergence (KLD). In dCA1, we found that the points in the scatter plots were closer to the unity line in APP/PS1 mice than in C57BL/6 mice (Fig 7B). This was reflected in the KLD, which was smaller in APP/PS1 mice, indicating a better agreement between the empirical and predicted pattern probabilities, than in C57BL/6 mice (Fig 7C). In vCA1, however, the KLD was higher, indicating a worse fit in the APP/PS1 animals than in the C57BL/6 animals (Fig 7D). These results, held true over a range of subpopulation sizes (Fig 7E and 7F) and showed that, in APP/PS1 mice, the functional interactions that shape macroscopic patterns of neuronal activity were differentially affected in dorsal and ventral CA1. Consistent with previous findings [49], relative to C57BL/6 mice, pairwise interactions were better able to predict the activity of the overall population in APP/PS1 mice in dCA1. On the other hand, activity patterns in APP/PS1 populations in vCA1 were less determined by those same pairwise interactions. Taken together, these results suggest that not only are the macroscopic and microscopic elements of network activity across the CA1 region of hippocampus differentially affected in the APP/PS1 model, but that the relationship between the microscopic interactions that govern correlations and the macroscopic features of global activity and network organization are altered by Aβ pathology.

**Fig 8:**
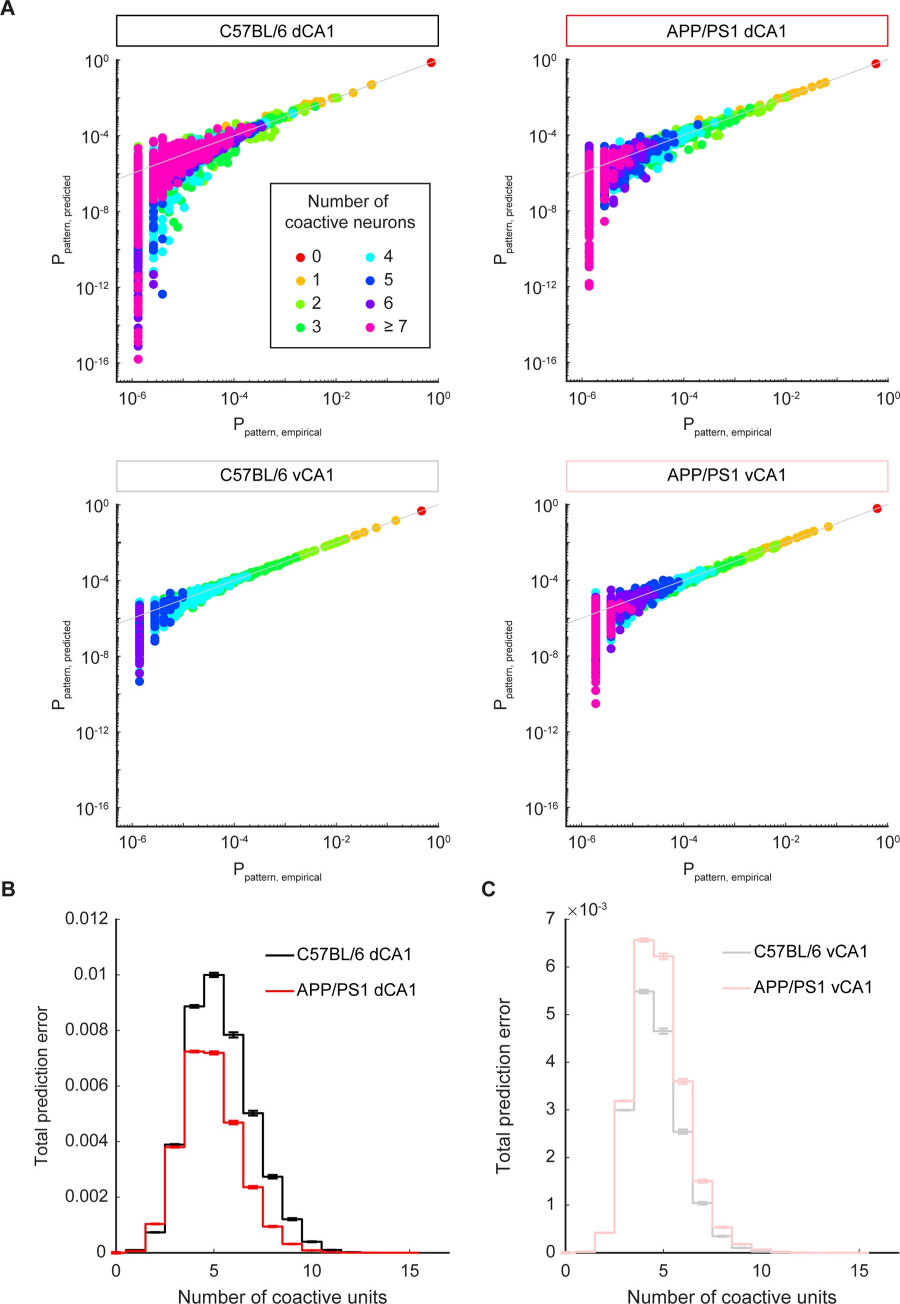
Disruptions in APP/PS1 population activity arise from disruptions to patterns with 4–5 coactive neurons. (A) Representative examples of maximum entropy model predicted probabilities and empirical probabilities, generated using 16-unit subpopulations. Each point denotes a single pattern and the grey line denotes unity. The color of the point corresponds to the number of coactive neurons in the pattern. (B) In dCA1, the total prediction error for APP/PS1 mice was lower than that for C57BL/6 mice across almost all pattern categories. The prediction error for patterns with 4–5 coactive units contributed most to the overall prediction error. Pattern probabilities were averaged across 2500 subsamples from 5 recording sessions in C57BL/6 mice and across 2000 subsamples from 4 recording sessions in APP/PS1 mice. These data were generated using 16-unit subpopulations. Error bars show the standard error of the mean. (C) In vCA1, the total prediction error for APP/PS1 mice was higher than that for C57BL/6 mice across almost all pattern categories. The prediction error for patterns with 4–5 coactive units contributed most to the overall prediction error. Pattern probabilities were averaged across 3000 subsamples from 6 recording sessions in C57BL/6 mice and across 4000 subsamples from 8 recording sessions in APP/PS1 mice. These data were generated using 16-unit subpopulations. Error bars show the standard error of the mean.

The KLD between the predicted and empirical pattern distributions provided a measure of the error of the maximum entropy model. However, it is averaged over a diverse array of population patterns. Different patterns had different numbers of co-activated neurons (Fig 6B). To understand whether certain categories of patterns were predicted less accurately by the maximum entropy models than others, we examined patterns by the number of coactive neurons in both CA1 subfields in C57BL/6 and APP/PS1 mice. First, we found that the gap between the empirical and predicted probabilities for patterns with many coactive neurons was higher than those with few active neurons (Fig 8A). To dissect how these variations in prediction error related to the KLD, we calculated the total prediction error for each pattern category, where category was defined by the total number of coactive neurons, regardless of the specific combinatorial pattern of that activity. As expected, individual patterns with larger numbers of coactive neurons had a higher per-pattern prediction error than those with lower numbers of coactive neurons (S10 Fig), likely reflecting higher order functional relationships between large groups of neurons beyond that which could be explained by pairwise interactions. However, the total prediction error, summed over all patterns in a given category, was greatest for patterns with 4–5 coactive neurons (Fig 8B and 8C). This was because the per-pattern prediction error for patterns with 4–5 coactive neurons (S10 Fig) was relatively poor *and* because this pattern category occurred more frequently in the empirical activity than categories with larger numbers of coactive neurons (Fig 6G and 6H). Only patterns with nonzero empirical probability were included in the KLD and error calculations. Thus, across both strains of mice and in both CA1 subfields, the largest contribution to the KLD came from the prediction error of patterns with 4–5 coactive neurons. For these patterns, and for most of the other categories, the total prediction error was lower in the APP/PS1 animals than the C57BL/6 animals in dCA1 (Fig 8B). This result was consistent with the decreased KLD in APP/PS1 mice in dCA1 (Fig 7C). In vCA1, the total prediction error curve was increased in APP/PS1 mice, relative to C57BL/6 mice (Fig 8C), consistent with the elevated KLD in APP/PS1 mice in vCA1 (Fig 7D). These findings indicate that in both dorsal and ventral CA1, the disruptions to population activity in APP/PS1 mice primarily arose from alterations in the coactivity of ensembles of 4–5 neurons. This was due to the fact that such patterns occur with sufficiently high empirical probability (Fig 6G and 6H) and that they have a sufficiently high per-pattern prediction error (S10 Fig). While the pattern prediction error was decreased in dCA1 in the APP/PS1 animals, it was increased in vCA1, suggesting that the effects of Aβ pathology percolated across the hippocampus in different ways.

**Fig 9:**
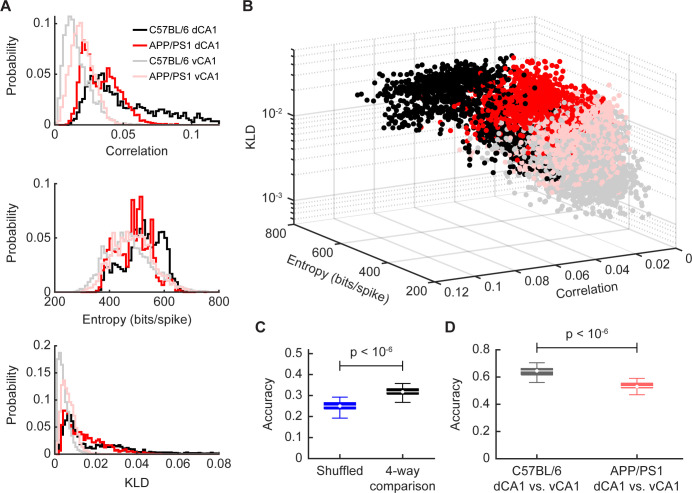
A signature of the differential impact of Aβ pathology on population activity is visible in the three-dimensional space defined by pairwise correlations, entropy, and the KLD of the maximum entropy model. (A) Distribution of correlation coefficients (top), entropy (middle), and KLD of the maximum entropy model (bottom) in dorsal and ventral CA1 in C57BL/6 and APP/PS1 mice (n_C57BL/6 dCA1_ = 2500 samples from 5 recording sessions, n_APP/PS1 dCA1_ = 2000 samples from 4 recording sessions, n_C57BL/6 vCA1_ = 3000 samples from 6 recording sessions, n_APP/PS1 vCA1_ = 4000 samples from 8 recording sessions). Each data point in these distributions represents the average over a 16-unit subsample. (B) Plot of correlations, entropy, and KLD of maximum entropy model for all recording sessions. Each point denotes a single resample (n_C57BL/6 dCA1_ = 2500 samples from 5 recording sessions, n_APP/PS1 dCA1_ = 2000 samples from 4 recording sessions, n_C57BL/6 vCA1_ = 3000 samples from 6 recording sessions, n_APP/PS1 vCA1_ = 4000 samples from 8 recording sessions). Each data point in these distributions represents the average over a 16-unit subsample. (C) Performance of decoder in a 4-way classification task to distinguish C57BL/6 dCA1, APP/PS1 dCA1, C57BL/6 vCA1, and APP/PS1 vCA1 samples (n = 100 iterations). (D) Performance of decoder in a 2-way classification task to distinguish C57BL/6 dCA1 from C57BL/6 vCA1 samples (n = 100 iterations) and in a 2-way classification task to distinguish APP/PS1 dCA1 from APP/PS1 vCA1 samples (n = 100 iterations), p < 10^−4^, two-sided Wilcoxon rank-sum test. Data in this figure were generated using 16-neuron subpopulations.

We have thus far examined the population code from three perspectives. The pair-wise correlations capture the interactions between the individual neurons, the entropy represents the diversity of patterns across the population, and the maximum entropy models reveal the extent to which the features of the network can be used to predict the global structure of the population. The distributions of each of these three properties for 16-unit subsamples varied between dCA1 and vCA1 as well as between C57BL/6 and APP/PS1 mice (Fig 9A). One can think of each of these measures as a feature of a high dimensional description of the population code, each of which can then be represented as a single dimension in a coding space. Thus, plotting the mean correlation, the entropy, and the KLD of the maximum entropy model for each ensemble of neuronal activity for an ensemble population of 16 neurons (black: C57BL/6 dCA1, gray: C57BL/6 vCA1, red: APP/PS1 dCA1, pink: APP/PS1 vCA1) allows us to see the relative position of the neural population code within this space (Fig 9B). It should be noted that these are not independent dimensions; populations with stronger pairwise correlations also had more patterns with multiple coactive units, as expected (Fig 5B,5C,6G and 6H). This, in turn, was associated with a larger entropy, given the increased diversity of patterns (Fig 6E and 6F), and poorer fit of the pairwise maximum entropy model, given the difficulty of predicting the occurrence of such highly coactive patterns using only pairwise interactions (Fig 7E and 7F). Nevertheless, this visualization revealed key insights. First, in the C57BL/6 mice, the dCA1 and vCA1 population clusters occupy different portions of the space, suggesting that the structure of the population code in dorsal and ventral CA1 is fundamentally different. Our analyses therefore reveal the consequences of the diverse anatomical and physiological properties of networks and circuits on the population code along the dorsal ventral axis of CA1 [13]. Perhaps more striking, however, was the effect that Aβ pathology has on this population code. The networks in the APP/PS1 animals resided in an entirely new part of the coding space, suggesting that Aβ pathology fundamentally altered all aspects of the population code. Second, the effects of Aβ pathology on population activity depended on whether that population was in dCA1 or vCA1. Finally, the dorsal and ventral CA1 clusters were closer to one another in the APP/PS1 mice than in the C57BL/6 animals.

To quantify these observations, we built a decoder to predict the strain identity and brain region of a subsample based on its population activity parameters (see Materials and Methods). The decoder performed significantly better than chance in a 4-way classification task (C57BL/6 dCA1 vs. APP/PS1 dCA1 vs. C57BL/6 vCA1 vs. APP/PS1 vCA1) (Fig 9C), suggesting that population activity can be used to distinguish both brain regions and mouse strains. Interestingly, however, the classifier was better able to distinguish dorsal and ventral CA1 in C57BL/6 mice than in APP/PS1 mice (Fig 9D). This suggests that in control mice, population activity varied across the longitudinal hippocampal axis, but that in APP/PS1 mice, this difference was degraded. In other words, Aβ pathology appears to have a homogenizing effect on population coding throughout the hippocampus, rendering the structure of network activity more similar in dorsal and ventral CA1. Taken together, these findings show that Aβ pathology distorts the geometry of the population code across the dorsoventral axis of the CA1 region of the hippocampus, and that the direction of that distortion varies depending on the hippocampal region.

**Fig 10:**
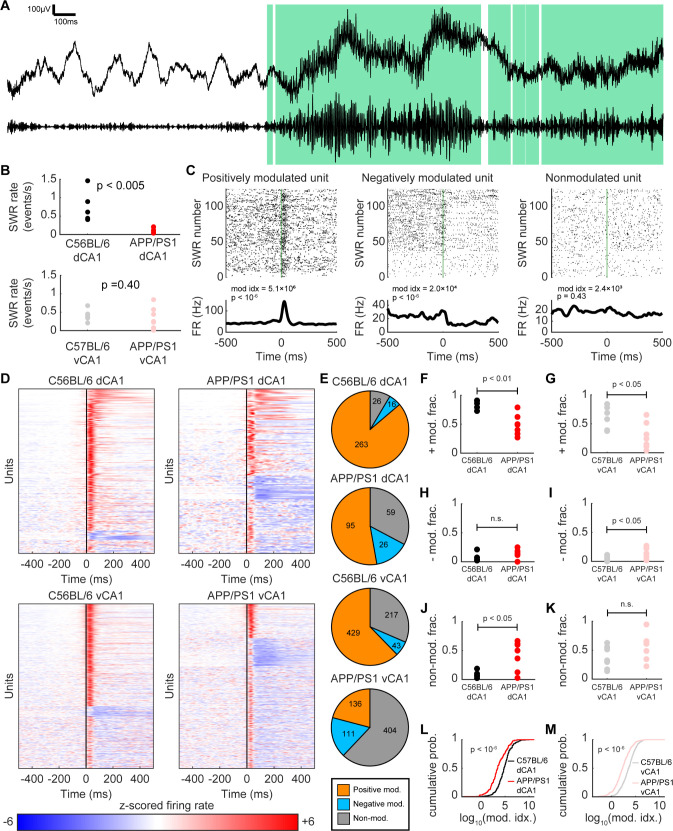
SWR-associated increase in neuronal firing rates is weakened in APP/PS1 mice in both dCA1 and vCA1. (A) Top: example raw widefield electrophysiological signal. Bottom: filtered signal band-passed at 150-250Hz. Green shaded regions denote SWR epochs. (B) Top: the abundance of SWRs in dCA1 was significantly decreased in APP/PS1 mice relative to C57BL/6 controls (mean ± std: C57BL/6 = 0.76 ± 0.43 events/s, APP/PS1 = 0.12 ± 0.07 events/s, p < 0.005, two-sided Wilcoxon sign-rank test, n_C57BL/6_ = 5 recording sessions from 4 animals, n_APP/PS1_ = 7 recording sessions from 5 animals). Each point denotes a recording session. Bottom: The abundance of SWRs in vCA1 was not significantly different between APP/PS1 mice and C57BL/6 controls (mean ± std: C57BL/6 = 0.41 ± 0.14 events/s, APP/PS1 = 0.31 ± 0.29 events/s, p = 0.40, two-sided Wilcoxon sign-rank test, n_C57BL/6_ = 7 recording sessions from 4 animals, n_APP/PS1_ = 8 recording sessions from 5 animals). Each point denotes a recording session. (C) Spiking activity from representative units that are positively modulated, negatively modulated, or non-modulated by SWRs. Top: Spike raster plots of each unit at the start of SWR epochs, with each row showing a different SWR event. Green line denotes the start of the SWR epoch. Bottom: peri-SWR time histogram (pSWR-TH) showing the averaged activity of each neuron across all SWR epoch starts in that recording session. (D) All pSWR-THs from all units in each strain and region. For visualization, units were first separated into positively modulated, negatively modulated, and non-modulated. Then, within each of these categories, units were sorted by modulation index. (E) Pie charts for each strain and region showing the proportion of positively modulated, negatively modulated, and non-modulated units. (F) The fraction of dCA1 units that were positively modulated by SWRs was significantly decreased in APP/PS1 mice relative to C57BL/6 controls (mean ± std: C57BL/6 = 0.84 ± 0.08, APP/PS1 = 0.49 ± 0.18, p < 0.01, two-sided Wilcoxon sign-rank test, n_C57BL/6_ = 5 recording sessions from 4 animals, n_APP/PS1_ = 7 recording sessions from 5 animals). (G) The fraction of vCA1 units that were positively modulated by SWRs was significantly decreased in APP/PS1 mice relative to C57BL/6 controls (mean ± std: C57BL/6 = 0.58 ± 0.20, APP/PS1 = 0.27 ± 0.22, p < 0.01, two-sided Wilcoxon sign-rank test, n_C57BL/6_ = 7 recording sessions from 4 animals, n_APP/PS1_ = 8 recording sessions from 5 animals). (H) The fraction of dCA1 units that were negatively modulated by SWRs were not significantly different between APP/PS1 mice and C57BL/6 controls (mean ± std: C57BL/6 = 0.07 ± 0.08, APP/PS1 = 0.10 ± 0.10, p > 0.99, two-sided Wilcoxon sign-rank test, n_C57BL/6_ = 5 recording sessions from 4 animals, n_APP/PS1_ = 7 recording sessions from 5 animals). (I) The fraction of vCA1 units that were negatively modulated by SWRs was significantly decreased in APP/PS1 mice relative to C57BL/6 controls (mean ± std: C57BL/6 = 0.05 ± 0.04, APP/PS1 = 0.16 ± 0.09, p < 0.05, two-sided Wilcoxon sign-rank test, n_C57BL/6_ = 7 recording sessions from 4 animals, n_APP/PS1_ = 8 recording sessions from 5 animals). (J) The fraction of dCA1 units that were non-modulated by SWRs was significantly increased in APP/PS1 mice relative to C57BL/6 controls (mean ± std: C57BL/6 = 0.09 ± 0.06, APP/PS1 = 0.41 ± 0.25, p < 0.05, two-sided Wilcoxon sign-rank test, n_C57BL/6_ = 5 recording sessions from 4 animals, n_APP/PS1_ = 7 recording sessions from 5 animals). (K) The fraction of vCA1 units that were non-modulated by SWRs was not significantly different between C57BL/6 and APP/PS1 mice (mean ± std: C57BL/6 = 0.37 ± 0.19, APP/PS1 = 0.57 ± 0.22, p = 0.07, two-sided Wilcoxon sign-rank test, n_C57BL/6_ = 7 recording sessions from 4 animals, n_APP/PS1_ = 8 recording sessions from 5 animals). (L) In dCA1, the SWR log_10_(modulation index) was significantly decreased in APP/PS1 mice relative to C57BL/6 controls (mean ± std: C57BL/6 = 4.84 ± 1.56, APP/PS1 = 3.61 ± 1.78 Hz, p < 10^−6^, two-sided Wilcoxon rank-sum test, n_C57BL/6_ = 305 neurons, n_APP/PS1_ = 180 neurons). (M) In vCA1, the SWR log_10_(modulation index) was significantly decreased in APP/PS1 mice relative to C57BL/6 controls (mean ± std: C57BL/6 = 3.50 ± 1.49, APP/PS1 = 2.28 ± 1.70 Hz, p < 10^−6^, two-sided Wilcoxon rank-sum test, n_C57BL/6_ = 689 neurons, n_APP/PS1_ = 651 neurons).

The entropy and maximum entropy models used here quantify the dimensionality of activity across large ensembles of neurons. One mechanism that has been proposed to orchestrate this collective neural activity is oscillations in the local field potential (LFP). A well-characterized feature of hippocampal LFPs is sharp waves and ripples (SWRs), brief 150-250Hz oscillations that typically occur during periods of rest or slow-wave sleep and have been posited to be involved in memory consolidation and future planning. Previous studies have reported variations in the occurrence of SWRs across the dorsal-ventral hippocampal axis [63] as well as disruptions in the frequency and power of SWRs in multiple mouse models of Alzheimer’s disease pathology [64,65]. We therefore sought to understand how the properties of SWRs and associated changes in single-unit activity across the hippocampal axis were altered in the context of Aβ pathology (Fig 10A). Consistent with prior results in other models of Alzheimer’s disease, we found that in dCA1, SWRs occurred less frequently in APP/PS1 mice than in C57BL/6 controls (Fig 10B). However, in vCA1, there was no significant difference in the occurrence of SWRs between the two strains (Fig 10B). Though this finding did not reflect the opposing directionality of Aβ-associated disruptions in dCA1 and vCA1 found in the correlation, entropy, and maximum entropy analyses, it does extend the idea that Aβ exerts heterogeneous effects across different brain regions to the level of regional LFP oscillations.

SWRs were also associated with consistent changes in the firing rate of individual units (Fig 10C). In C57BL/6 and APP/PS1 mice across both dCA1 and vCA1, neurons that were positively-modulated, negative-modulated, and non-modulated in the context of SWRs were identified (Fig 10D), though the frequency of each of these subpopulations varied across strains and brain regions (Fig 10E). In both dCA1 and vCA1, the fraction of neurons that were positively modulated by SWRs was decreased in APP/PS1 mice, relative to C57BL/6 controls (Fig 10F and 10G). Additionally, the fraction of neurons that were negatively modulated by SWRs was increased in APP/PS1 mice in vCA1, though this effect was not seen in dCA1 (Fig 10H and 10I). Finally, the fraction of neurons with firing rates unchanged during SWRs was increased in APP/PS1 mice in dCA1, but no change was observed in vCA1 (Fig 10J and 10K). We quantified the magnitude of the change in single unit firing rates by calculating the modulation index [66]. In both dCA1 and vCA1, the modulation index was decreased in the APP/PS1 mice, relative to C57BL/6 control mice (Fig 10L and 10M). Taken together, these findings suggest that the orchestration of neuronal activity by SWRs is weakened in APP/PS1 mice across the hippocampus. Future analyses of variations in population-level features (e.g., correlations, entropy) in the context of SWRs (S11 Fig) or other hippocampal LFP oscillations, such as theta or gamma, could further elucidate how the relationship between different scales of neural activity is disrupted by Aβ pathology [9].

## Discussion

We recorded the activity of large neuronal populations in awake C57Bl/6 and APP/PS1 mice across dorsal and ventral CA1 as the animals navigated virtual reality environment. Consistent with previous studies that examined spontaneous population activity in dCA1 in APP/PS1 animals [49], key markers of population activity, such as correlations, entropy, and prediction error of a pairwise maximum entropy model, were reduced in APP/PS1 mice between 14–19 months of age while they were in a virtual reality environment. These suggest that the changes observed are a general feature of the Aβ pathology on circuits in dCA1. In vCA1, however, we found increased correlations, increased entropy, and a greater error in the maximum entropy model in APP/PS1 mice as compared to age matched control animals. These results show a differential impact of Aβ pathology on dorsal versus ventral CA1. Taken together, they suggest that the impact of AD on population activity is not simply a function of Aβ burden, but also a product of the interplay between histopathology and the properties of the neurons and circuits in each region.

The divergent impacts on population coding observed in the APP/PS1 mice across these two subregions could arise from multiple sources. First Aβ pathology could have a different effect on the neurons and intrinsic circuits of dCA1 as compared to those of vCA1 because these circuits are different. Differences across the longitudinal axis of the CA1 region include variations in gene expression [17,67], differences in neuronal morphology, and different biophysical properties in the CA1 pyramidal cells [68,69]. For example, vCA1 neurons have lower dendritic length, decreased apical dendritic branching, higher membrane potential, and higher input resistance than dCA1 neurons [68,69]. As a result of these differences, the structure of population activity would be different, something we found in this work. A specific pathology then, such as Aβ accumulation, would then exert divergent effects on different parts of the hippocampus because the networks in these regions are functionally different.

A second possibility is that Aβ pathology differentially impacts the brain regions that project to either dorsal or ventral CA1. There exist substantial variations in afferent innervation throughout the longitudinal hippocampal axis. For example, there is a topographic organization of entorhinal cortex (EC)-hippocampus circuits, such that dorsal hippocampus preferentially receives projections from dorsolateral EC, while ventral hippocampus preferentially receives input from ventromedial EC [70–72]. In turn, each of these EC subregions have their own distinct patterns of afferent connectivity; for example, dorsal and lateral EC receive inputs from anterior cingulate and retrosplenial cortex while ventral and medial EC receive inputs from infralimbic and prelimbic cortex [73–75]. In addition, a recent study of direct whole-brain inputs to CA1 projection neurons (PNs) showed that vCA1 PNs receive a more diverse array of inputs from throughout the brain, including the basolateral amygdala and piriform cortex, than dCA1 PNs, whose inputs primarily arise from the EC and other hippocampal subfields [76]. Given the variations in Aβ burden across different brain regions [77,78], it is possible that the differences in population activity between dorsal and ventral CA1 are inherited from upstream circuits.

Our work suggests that the differences in network activity that exist across the longitudinal axis of the hippocampus (the initial conditions of the circuit) are differentially altered by the Aβ accumulation in the APP/PS1 animals. A natural question is how might a single pathology exert such heterogeneous, often opposite effects on the networks of the hippocampus? Evidence suggests that the effects of Aβ on neurons and synapses can be heterogeneous, with the precise nature of the disruption complex and contingent on many factors [33,79]. For example, moderate elevations in Aβ appear to facilitate synaptic potentiation (including long-term potentiation (LTP)) by increasing presynaptic release probabilities [80,81], while large increases in Aβ levels lead to decreased LTP and enhanced long term depression (LTD) by altering post-synaptic mGluR and NMDAR signaling [81,82]. Coupled with findings of differences in the induction and magnitude of LTP and LTD between dorsal and ventral CA1 neurons [83–86], these observations hint at the possibility that Aβ pathology may differentially affect synaptic plasticity in dorsal and ventral hippocampus. Multiple studies have shown that Aβ pathology was associated with deficits in the induction or maintenance of LTP in the dorsal hippocampus [87–89]. Changes in synaptic coupling due to the differential effects of Aβ pathology in the APP/PS1 mice could induce either LTP or LTD across different cell types and different regions of the hippocampus, leading to diverse impacts on the correlations that would arise from changes in synaptic strength. Furthermore, studies showing differences in the intrinsic excitability of neurons in mouse models of Aβ pathology [90] also suggest that the biophysical properties of neurons that influence spike-timing dependent plasticity [91–93] could either increase or decrease correlations depending on whether Aβ renders those neurons more excitable or less excitable. In this regard, the diametric effects of Aβ pathology in the APP/PS1 animals on correlations, entropy, and the structure of population activity may reflect the heterogeneous effects of that pathology on individual neurons and synapses.

We consider what this result might tell us about how network activity determines behavior. In patients with AD, many cognitive domains (episodic memory, spatial cognition, etc.) are blunted [94–98], while other domains, (stress, anxiety, etc.), have been shown to be enhanced [99–102]. The findings of this study offer one insight into a potential mechanism behind these disparate effects. In an ensemble coding scheme, wherein each pattern represents a particular percept or memory [58,59], a reduction in entropy or in pairwise correlations would erode representations, something that might give rise to loss in a cognitive or mnemonic domain. Such degradations in population activity have been suggested as the basis for perceptual and cognitive deficits in other mouse models of psychiatric disease [46,103]. By contrast, increases in correlations and entropy may provide a neural substrate for enhanced anxiety or fear. Correlations and increased entropy could be thought of as increases in generalization, which would, for example, alter how sensory experiences are mapped onto anxiety. Our results suggest that understanding the behavioral changes in AD may emerge not only by looking at cellular pathology, but the diverse impact that pathology has on network function including the ways in which neuronal population activity is altered.

## Materials and methods

### Ethics statement

The experiments performed in this study were approved by the Institutional Animal Care and Use Committee at the University of Rochester.

### Mice

All experiments were performed in accordance with regulatory standards and were approved by the Institutional Animal Care and Use Committee (IACUC) at the University of Rochester. Six male APP/PS1 mice (Strain #034832,The Jackson Laboratory, Bar Harbor, ME) and four male C57BL6/J mice (Strain #000664, The Jackson Laboratory) were included in the study. The APP/PS1 mice expressed a chimeric mouse/human amyloid precursor protein with the APP695swe mutation as well as a mutant human presenilin 1 PS1-dE9 [104]. At the time of recording, mice were 14 to 19 months of age. Surgeries and recordings were randomized to ensure that no systematic biases were introduced. Mice were housed in transparent cages on a 12h/12h light/dark cycle. All recording were performed in the light phase. Mice were not used for any previous experiments or procedures.

### Virtual reality setup

A one-dimensional virtual track was generated using the Virtual Reality MATLAB (VirMEn) toolbox based on previously published designs [27,28,44]. The virtual track was projected onto a curved board, which occupied 180° of the visual field of the mouse. A rotational encoder in the axel of the run-wheel transmitted wheel movement information to the computer, which updated the position of the animal in the virtual track accordingly. Upon reaching the end of the virtual track, mice received a small sweetened milk reward from a lick spout and their position was then updated to the beginning of the track. To minimize ambient light, sound and electromagnetic interference, the rig was enclosed in an electrically shielded box.

### Head fixing

Prior to surgery, animals were anesthetized using a 1–2% isoflurane mixture and placed in a stereotactic surgical rig. The scalp was resected, scored, and a craniotomy sites for dCA1 (coordinates relative to bregma: 2.5mm caudal, 1.5mm right) and vCA1 (coordinates relative to bregma: 3.05mm caudal, 3.15mm right) were marked. Next, the skull was scored and a metal ground pin and 3D-printed PLA headframe were attached to the skull using dental cement (Ortho-Jet Powder and Jet Liquid, Lang Dental Manufacturing Company, Wheeling, IL) and veterinary adhesive (Vetbond, The 3M Company, Maplewood, MN). Post-surgical analgesia was provided for 72h using a subcutaneous injection of 0.5–1.0 mg/kg slow-release buprenorphine, in accordance with IACUC protocols.

### Run training

Animals were given one day to recover following headframe implantation surgery. Subsequently, a 7-day training period was started to habituate the mice to the run-wheel, the virtual reality environment, and the lick spout [105]. Each day, mice were head-fixed in the virtual environment and allowed to run on the wheel for one hour. No electrophysiological recordings were performed during this habituation phase.

### Craniotomy

Animals were anesthetized using a 1–2% isoflurane mixture. A craniotomy was performed over dCA1 and vCA1 using the sites marked during the headframe implantation surgery. A silicone sealant (Kwik-Cast, World Precision Instruments, Sarasota, FL) was applied over the craniotomy sites for protection. After the surgery, mice were allowed to recover for 12–18 hours in their home cages before electrophysiological recordings. This recovery period has previously been shown to not affect animal behavior [105].

### Electrophysiology

Extracellular voltage recordings were performed using an open source microfabricated silicon electrode array [106,107] comprised of 128 recording channels spread across four shanks, each of which was spaced 50 μm apart. The probes were coated with a fluorescent dye (Alexa Fluor, Thermo Fisher Scientific, Waltham, MA) and connected to an Intan RHD 128-channel headstage and RHD USB interface board (Intan Technologies, Los Angeles, CA).

After the recovery period, animals were head-fixed into the run-wheel in the virtual reality rig. The electrode array was placed on a stereotactic frame and guided to the location of the craniotomies. For dCA1, the probe was lowered 1–1.2 mm from the brain surface and for vCA1, the probe was lowered 4–4.2 mm from the brain surface. Two days of recordings were performed for each mouse. For most animals, each day of recording comprised of one 1-2h session in dCA1 and one 1-2h session in vCA1. Signals were acquired at 30kHz in the 0.1–3500 Hz frequency band.

### Spike sorting

Preprocessing and spike sorting was performed using the open-source toolbox Kilosort 2 [29]. The widefield raw electrophysiology data were high passed at 500Hz, the median signal from all channels was subtracted from each stream, and correlated noise across all channels was removed. A set of template waveforms and spike times was generated and iteratively updated to reconstruct the original data set. The putative single units that resulted from this process were manually curated using the visualization software Phy 2 [30]. Based on the waveforms, amplitudes, and inter-spike interval distributions, units were preserved, eliminated, or merged. Units without clear refractory periods were eliminated. Additionally, the physical location of each unit in the brain was estimated based on the relative magnitude of its action potentials across different channels. After spike sorting, the data were imported into MATLAB 2019a (The MathWorks, Inc., Natick, MA) for analysis of population activity. Units were classified as excitatory or inhibitory based on the shape of the mean waveform [31,32]. Excitatory units were identified as those with a trough-to-peak time of over 0.5ms, while inhibitory units were those with a trough-to-peak time of less than 0.5ms.

### Spatial information

The virtual track was divided into 111 bins, each of size ~1.7cm. For a given unit, the mean firing rate in each bin was calculated by dividing the number of spikes in each bin by the amount of time the animal spent in that bin. This map was then smoothed by convolving with a 5-bin-wide square wave. Only intervals during which the animal’s velocity exceeded 5 cm/s were included for this analysis. The spatial information was calculated with the following formula [108]:

$$I_{single-unit}=\sum_{x}{-p}_{x}\lambda_{x}\log_{2}\frac{\lambda }{\lambda_{x}}$$ (1)

In this equation, *p*_*x*_ denotes the probability that the animal is occupying spatial bin *x*, *λ*_*x*_ denotes the firing rate in bin *x*, and *λ* denotes the mean firing rate across all bins.

### Spike train circular shuffling

Shuffled spike trains were generated to calculate spatial information null distributions. All of the spikes from each neuron were shifted by random interval. If a shifted spike time exceeded the duration of the recording, the modulus of the spike time by the recording duration was used instead, such that out-of-bounds spikes were wrapped around to the beginning of the spike train. For each neuron, this process was repeated 100 times. The spatial information of the circularly shuffled spike trains was then calculated using Formula (1).

### Spatial stability score

To quantify the stability of spatial tuning across the duration of recording session, the recording session was split into halves, and smoothed firing rate maps were generated for each half. For a given unit, the stability score was defined as the correlation coefficient between the firing rate maps for the two halves, as has been done in previous studies of spatial coding [38,39].

### Firing rate variance and bootstrap sampling

To compare the heterogeneity of mean firing rates between different groups and brain regions, hypothesis testing was performed, with the ratio of the firing rate variances between the two groups ($\frac{\sigma_{A}^{2}}{\sigma_{B}^{2}}$) serving as the test statistic. As normality could not be assumed, a bootstrap sampling approach with n = 10,000 resamples with replacement was used to estimate null distributions of the test statistic. The p-value was defined as the proportion of bootstrap samples for which the ratio of variances exceeded 1.0. If p < 0.025 or p > 0.975, the null hypothesis (that the ratio of variances is 1.0) was rejected.

### Firing rate correlations and graph properties

For each unit, a time-dependent firing rate trace was obtained by calculating the number of spikes in a sliding 10ms window and converting the resulting time-series to a z-score. The correlation coefficient between each pair of traces was calculated and visualized as a correlation matrix. To ensure that the mean correlation values were not disproportionately weighted towards recording sessions with high neuron counts, a random subsample of 250 unit pairs was taken from each session. The matrices were converted to graph representations, where each neuron was represented by a node [53]. A threshold was applied to the matrices, such that any correlation value that exceeded the threshold was preserved as an edge between the two corresponding nodes, while edges that fell below the threshold were discarded. The relative degree was calculated by summing the number of edges in the graph and dividing by the total number of possible edges between all the nodes in the graph. The clustering coefficient was based on node triplets, defined as three nodes that are connected to each other by either two edges (open triplet) or three edges (closed triplet). The clustering coefficient was defined as the proportion of all node triplets that are closed triplets. To ensure that the graphs being compared were of the same size, networks of 16-neuron subsamples were constructed for each recording session. Graph measures were calculated using the MATLAB Brain Connectivity Toolbox [109].

### Entropy

Spiking activity for each unit was binned into 10ms non-overlapping bins, such that a value of 1 would be assigned to the bin if at least one spike occurred in that 10ms interval, and a value of 0 would be assigned if no spikes occurred. When this was done for all the units in a given animal, a matrix of binned activity was generated. A single column of this matrix thus represented the activity of all the neurons of the population within a 10ms window, which we refer to as a pattern [28,54,110]. In a population of 10 units, there would thus be 2^10^ = 1024 possible binary patterns. The entropy of the population, which quantifies the diversity of observed patterns, can be described with the following formula, where *k* denotes a particular pattern and *p*_*k*_ denotes the probability of that pattern:

$$\sum_{k}{-p}_{k}\log_{2}p_{k}$$ (2)

[110]. This number is then normalized by dividing by the mean firing rate of the population.

### Maximum entropy modeling

Fitting of maximum entropy models was performed using the maxent_toolbox [111]. The central principle of a maximum entropy model is the generation of a pattern probability distribution in which the activity of individual neurons and the coactivity of pairs of neurons match those of empirical probability distribution, but for which there is no further structure. More concretely, a maximum entropy model is a set of terms *h*_*i*_, which describe the average activity of individual neurons, and *J*_*ij*_, which describe the functional coupling between pairs of neurons. Given these terms, a pattern probability distribution for a neuronal population can be generated using the following:

$$P_{predicted}(\sigma_{1},\sigma_{2}{\ldots \sigma }_{n})=\frac{1}{Z}e^{\sum_{i}h_{i}\sigma_{i}+\frac{1}{2}\sum_{i\neq j}J_{ij}\sigma_{i}\sigma_{j}}$$ (3)

in which *σ*_*i*_ denotes the binary state of neuron *i*, *P*_*predicted*_ (*σ*_1_,*σ*_2_…*σ*_*n*_) denotes the predicted probability of a particular pattern, and Z indicates the partition function used to normalize the probability distribution. The objective is to iteratively adjust the terms *h*_*i*_ and *J*_*ij*_ such that they not only match the above constraints, but also result in a predicted pattern probability distribution with the least amount of structure (or alternatively, maximum entropy). The resultant predicted pattern probabilities thus represent the expected behavior of a population of neurons in the absence of any higher-order interactions beyond pairwise couplings [44,54].

The fit between the model prediction and the empirical pattern probabilities were quantified using the Kullback-Leibler Divergence (KLD) and the prediction error. For a given pattern, the prediction error was defined as follows:

$$\frac{\left|p_{k,predicted}-p_{k,empirical}\right|}{p_{k,empirical}}$$ (4)

in which *p*_*k*,*predicted*_ and *p*_*k*,*empirical*_ denote the predicted and empirical probabilities of a given pattern, respectively. The prediction errors for individual patterns were then summed across all patterns with a given number of coactive units:

$${Total\;prediction\;error}_{C}=\sum_{k|(k\in C\;and\;p_{k,empirical}\neq 0)}\frac{{|p}_{k,predicted}-p_{k,empirical}|}{p_{k,empirical}}$$ (5)

in which *C* denotes the set of all patterns with a given number of coactive units. The mean per-pattern prediction error was defined as the total prediction error divided by the number of patterns in *C* that occurred with a nonzero empirical probability:

$${Mean\;per\;pattern\;prediction\;error}_{C}=\frac{{Total\;prediction\;error}_{C}}{n(k|(k\in C\;and\;p_{k,empirical}\neq 0))}$$ (6)

In which *n* denotes the cardinality of the contained set. To align with the KLD, only patterns that occurred with a nonzero empirical probability were included in the prediction error calculations.

To ensure sufficiently large populations, for the correlation, entropy, and maximum entropy calculations, only recording sessions with over 16 neurons were included. For each session, the neuronal population was repeatedly sampled to generate subpopulations of 16 neurons. The entropy, maximum entropy fit, and pattern probabilities of each subsample was calculated to generate a distribution of values for each recording session.

### Decoding brain region and strain identity from population activity

16-neuron subpopulations were generated for each recording session. For each subsample, the mean pairwise correlation, the entropy, and the KLD of the maximum entropy model were calculated; these were visualized in a 3D scatter plot. The data were then randomly split into training (80%) and testing (20%) subsets and used for three different classification tasks. The objective for the first task was to predict the brain region and strain identity (C57BL/6 dCA1 vs. APP/PS1 dCA1 vs. C57BL/6 vCA1 vs. APP/PS1 vCA1) for each point in the test set. For each point in the test set, the 5 nearest neighbors in the training set were identified; the mode identity of these 5 nearest neighbors was assigned as the predicted identity of the test set point. Accuracy was defined as the fraction of test points with correctly predicted identities. This process was repeated 100 times with different train/test set splits. A null distribution was generated by repeating this procedure after shuffling the identities of the points in the training set. The objective for the second and third tasks, respectively, was to predict the brain region for the C57BL/6 mice only (C57BL/6 dCA1 vs. C57BL/6 vCA1) and for the APP/PS1 mice only (APP/PS1 dCA1 vs. APP/PS1 vCA1).

### Sharp waves/ripples analyses

Sharp waves/ripple (SWR) epochs were detected using previously described methods [63,65]. For each channel, the wide band signal was band pass filtered at 150-250Hz. This signal was then squared and summed across all the channels in a recording session and subsequently smoothed with a 1D Gaussian kernel of standard deviation 4ms and width 32ms. SWR epochs were identified as intervals during which this averaged and smoothed signal exceeded 2 standard deviations from the mean. Those that were shorter than 15ms, those that started less than 1s after the end of the prior SWR, or those that occurred when the animal was locomoting were eliminated.

To identify units whose firing rates were modulated by SWRs, a peri-SWR time histogram (pSWR-TH) was calculated from 500ms prior to the start of the SWR to 500ms after the start of the SWR. For each unit, the spikes in this window were circularly shuffled 500 times and a mean shuffled pSWR-TH was calculated. A SWR modulation index [66] was defined as the squared difference between the empirical pSWR-TH and the mean shuffled pSWR-TH in the 200ms window after the start of the SWR. The significance of this modulation index was determined by generating a null distribution with the 500 shuffled pSWR-THs; a unit was considered to be significantly modulated by SWRs if the empirical modulation index exceeded the 95^th^ percentile of this null distribution. For all SWR-modulated units, the modulation lag was defined as the time from the start of the SWR epoch to the time corresponding to the extremum of the pSWR-TH. For the plots in Fig 10C and 10D, the pSWR-TH was smoothed using a Gaussian of width 80ms and standard deviation 10ms.

To compare neural activity during SWR epochs and non-SWR epochs, we generated two sets of spike trains for each recording session. For the first spike train, we concatenated all the spikes that occurred in the 500ms interval following the start of each SWR event. Then, for each SWR event, we identified an 500ms interval in the recording that did not contain an SWR. We concatenated the spikes that occurred in all of these intervals to generate the second spike train. We then calculated the pairwise correlations for each set of spike trains, as described above.

### Tissue processing and staining

Transcardial perfusion was performed first with PBS solution, followed by a 4% paraformaldehyde (PFA) solution. The brain was removed from the skull, placed in a 4% PFA solution for 24 hours, then transferred to a 30% sucrose-PFA solution for 48 hours. Brains were frozen, sliced into 100μm coronal sections, mounted using Hoechst-containing media (Hoechst 33258, Invitrogen), and cover-slipped. For sections that contained the hippocampus (1.25mm to 3.5mm posterior to bregma), after every four 100μm sections, a single 26μm coronal section was taken. These 26μm sections were stained for Aβ plaques with Congo Red (HT60-1KT, Milipore Sigma, Burlington, MA). Briefly, the sections were incubated in a 1:100 NaOH/NaCl solution for 20 minutes, stained with a 1:100 NaOH/Congo Red solution for 20 minutes, and rinsed several times with PBS before being mounted with Hoechst-containing media and cover-slipped.

### Slide imaging and scanning

The hippocampal sections stained with Congo Red were imaged using an Olympus VS120 slide scanner (Olympus Corporation, Tokyo, Japan).

### Aβ plaque quantification

The images were imported into an image analysis software Qupath [112], where Aβ plaque boundaries were manually circumscribed and hippocampal subfield boundaries were delineated. These data were then imported into MATLAB, where plaque numbers and fractional plaque area were computed for dorsal and ventral hippocampus and related to the electrophysiological properties of those respective regions.

## Supporting information

S1 Fig Spike waveforms were used to separate inhibitory and excitatory neurons. Histogram shows distribution of trough-peak times for all recorded neurons. A clear bimodal distribution was found and a threshold of 0.5ms was chosen to distinguish cell classes (n_excitatory_ = 1197 units, n_inhibitory_ = 628 units).
(EPS)

S2 Fig No significant correlations between Aβ plaque burden and mean firing rate of excitatory neurons or inhibitory neurons in either hippocampal subfield (A) There was no significant correlation between the mean firing rate of dCA1 excitatory neurons and the dCA1 Aβ plaque density (r = -0.20, p = 0.78, Spearman rank correlation coefficient, n = 5 animals). Each point denotes a single animal. The black solid line denotes the least-squares regression and the black dashed lines denote the boundaries of the 95% confidence interval of the regression. (B) There was no significant correlation between the mean firing rate of vCA1 excitatory neurons and the vCA1 Aβ plaque density (r = 0.37, p = 0.50, Spearman rank correlation coefficient, n = 6 animals). Each point denotes a single animal. The grey solid line denotes the least squares regression and the grey dashed lines denote the boundaries of the 95% confidence interval of the regression. (C) There was no significant correlation between the mean firing rate of dCA1 inhibitory neurons and the dCA1 Aβ plaque density (r = -0.60, p = 0.35, Spearman rank correlation coefficient, n = 5 animals). Each point denotes a single animal. The black solid line denotes the least-squares regression and the black dashed lines denote the boundaries of the 95% confidence interval of the regression. (D) There was no significant correlation between the mean firing rate of vCA1 inhibitory neurons and the vCA1 Aβ plaque density (r = 0.60, p = 0.24, Spearman rank correlation coefficient, n = 6 animals). Each point denotes a single animal. The grey solid line denotes the least squares regression and the grey dashed lines denote the boundaries of the 95% confidence interval of the regression.
(EPS)

S3 Fig Spatial firing rate maps of representative recording sessions from C57BL/6 and APP/PS1 mice in dCA1 and vCA1. The activity of each neuron was scaled such that the bins with the minimum and maximum activity corresponded to 0 and 1, respectively.
(EPS)

S4 Fig Spatial stability is decreased in APP/PS1 mice in both dCA1 and vCA1. (A) In dCA1, stability score was significantly decreased in APP/PS1 mice relative to C57BL/6 mice (mean ± std: C57BL/6 = -0.009 ± 0.292, APP/PS1 = -0.1845 ± 0.256, p < 10^−6^, two-sided Wilcoxon rank-sum test, n_C57BL/6_ = 295 units from 5 recording sessions, n_APP/PS1_ = 167 units from 4 recording sessions). (B) In vCA1, stability score was significantly decreased in APP/PS1 mice relative to C57BL/6 mice (mean ± std: C57BL/6 = -0.069 ± 0.285, APP/PS1 = -0.165 ± 0.285, p < 10^−6^, two-sided Wilcoxon rank-sum test, n_C57BL/6_ = 613 units from 6 recording sessions, n_APP/PS1_ = 645 units from 8 recording sessions).
(EPS)

S5 Fig Spatial information of excitatory neurons in APP/PS1 mice are decreased in dCA1 and vCA1. (A) In dCA1, spatial information was decreased in APP/PS1 mice relative to C57BL/6 controls (mean ± std: C57BL/6 = 0.134 ± 0.050, APP/PS1 = 0.132 ± 0.054, p < 0.01, two-sided Wilcoxon rank-sum test, n_C57BL/6_ = 229 units from 5 recording sessions, n_APP/PS1_ = 124 units from 4 recording sessions). The spatial information in dCA1 was significantly larger than circularly shuffled spike trains with similar mean firing rates for C57BL/6 mice (mean ± std: empirical = 0.134 ± 0.050, shuffled = 0.123 ± 0.035, p < 0.005, two-sided Wilcoxon rank-sum test, n_empirical_ = 229 units from 5 recording sessions, n_shuffled_ = 22900 simulated units from 5 recording sessions), but not for APP/PS1 mice (mean ± std: empirical = 0.132 ± 0.054, shuffled = 0.124 ± .054, p = 0.13, two-sided Wilcoxon rank-sum test, n_empirical_ = 124 units from 4 recording sessions, n_shuffled_ = 12400 simulated units from 4 recording sessions). (B) In vCA1, spatial information was decreased in APP/PS1 mice relative to C57BL/6 controls (mean ± std: C57BL/6 = 0.143 ± 0.069, APP/PS1 = 0.115 ± 0.039, p < 10^−6^, two-sided Wilcoxon rank-sum test, n_C57BL/6_ = 450 units from 6 recording sessions, n_APP/PS1_ = 394 units from 8 recording sessions). The spatial information in vCA1 was significantly larger than circularly shuffled spike trains with similar mean firing rates for C57BL/6 mice (mean ± std: empirical = 0.143 ± 0.069, shuffled = 0.125 ± .049, p < 0.001, two-sided Wilcoxon rank-sum test, n_empirical_ = 450 neurons from 6 recording sessions, n_shuffled_ = 45000 simulated neurons from 6 recording sessions), but not for APP/PS1 mice (mean ± std: empirical = 0.115 ± 0.039, shuffled = 0.110 ± 0.035, p = 0.18, two-sided Wilcoxon rank-sum test, n_empirical_ = 394 neurons from 8 recording sessions, n_shuffled_ = 39400 simulated neurons from 8 recording sessions).
(EPS)

S6 Fig Similar firing rates between C57BL/6 and APP/PS1 mice. (A) In dCA1, overall firing rates in C57BL/6 and APP/PS1 mice were not significantly different (mean ± std: C57BL/6 = 4.4 ± 5.3 Hz, APP/PS1 = 5.4 ± 7.3 Hz, p = 0.43, two-sided Wilcoxon rank-sum test, n_C57BL/6_ = 305 neurons, n_APP/PS1_ = 180 neurons). (B) In vCA1, overall firing rates in C57BL/6 and APP/PS1 mice were not significantly different (mean ± std: C57BL/6 = 5.5 ± 9.5 Hz, APP/PS1 = 4.9 ± 7.8 Hz, p = 0.78, two-sided Wilcoxon rank-sum test, n_C57BL/6_ = 689 neurons, n_APP/PS1_ = 651 neurons). (C) In C57BL/6 animals, overall firing rates were similar in dCA1 and vCA1 mean ± std: dCA1 = 4.4 ± 5.3 Hz, vCA1 = 5.5 ± 9.5 Hz, p = 0.42, two-sided Wilcoxon rank-sum test, n_dCA1_ = 305 neurons, n_vCA1_ = 689 neurons). (D) In dCA1, excitatory neuron firing rates in C57BL/6 and APP/PS1 mice were not significantly different (mean ± std: C57BL/6 = 3.5 ± 4.4 Hz, APP/PS1 = 3.3 ± 3.1 Hz, p = 0.43, two-sided Wilcoxon rank-sum test, n_C57BL/6_ = 229 neurons, n_APP/PS1_ = 88 neurons). (E) In vCA1, excitatory neuron firing rates in C57BL/6 and APP/PS1 mice were not significantly different (mean ± std: C57BL/6 = 2.5 ± 3.1 Hz, APP/PS1 = 2.5 ± 2.7 Hz, p = 0.36, two-sided Wilcoxon rank-sum test, n_C57BL/6_ = 450 neurons, n_APP/PS1_ = 397 neurons). (F) In C57BL/6 mice, firing rates were significantly higher for excitatory neurons in dCA1 than those in vCA1 (mean ± std: dCA1 = 3.5 ± 4.4 Hz, vCA1 = 2.5 ± 3.1 Hz, p < 10^−3^, two-sided Wilcoxon rank-sum test, n_dCA1_ = 229 neurons, n_vCA1_ = 450 neurons). (G) In dCA1, inhibitory neuron firing rates in C57BL/6 and APP/PS1 mice were not significantly different (mean ± std: C57BL/6 = 7.2 ± 6.7 Hz, APP/PS1 = 10.4 ± 10.7 Hz, p = 0.16, two-sided Wilcoxon rank-sum test, n_C57BL/6_ = 76 neurons, n_APP/PS1_ = 56 neurons). (H) In vCA1, inhibitory neuron firing rates in C57BL/6 and APP/PS1 mice were not significantly different (mean ± std: C57BL/6 = 11.2 ± 13.9 Hz, APP/PS1 = 8.7 ± 10.9 Hz, p = 0.24, two-sided Wilcoxon rank-sum test, n_C57BL/6_ = 239 neurons, n_APP/PS1_ = 257 neurons). (I) In C57BL/6 mice, firing rates were not significantly different for inhibitory neurons in dCA1 and those in vCA1 (mean ± std: dCA1 = 7.2 ± 6.7 Hz, vCA1 = 11.2 ± 13.9 Hz, p = 0.53, two-sided Wilcoxon rank-sum test, n_dCA1_ = 76 neurons, n_vCA1_ = 239 neurons).
(EPS)

S7 Fig Increased heterogeneity of neural activity in vCA1 relative to dCA1 in C57BL/6 mice. The variance of neuronal firing rates were compared across regions and groups and null distributions were generated using a bootstrap sampling approach. These null distributions were in turn used to calculate confidence intervals and p-values for hypothesis testing. (A) In C57BL/6 mice, the firing rate variance was significantly larger in vCA1 than in dCA1 (σ^2^_C57BL/6 dCA1_ = 27.8 Hz^2^, σ^2^_C57BL/6 vCA1_ = 90.0 Hz^2^, σ^2^_C57BL/6 dCA1_/σ^2^_C57BL/6 vCA1_ 95% CI = 0.18–0.49, p < 10^−6^, n = 10,000 resamples with replacement). (B) There was no significant difference in firing rate variance in dCA1 between C57BL/6 and APP/PS1 mice (σ^2^_C57BL/6 dCA1_ = 27.8 Hz^2^, σ^2^_APP/PS1 dCA1_ = 53.0 Hz^2^, σ^2^_C57BL/6 dCA1_/σ^2^_APP/PS1 dCA1_, 95% CI = 0.28–1.09, p = 0.04, n = 10,000 resamples with replacement). (C) There was no significant difference in firing rate variance in vCA1 between C57BL/6 and APP/PS1 mice (σ^2^_C57BL/6 vCA1_ = 90.0 Hz^2^, σ^2^_APP/PS1 vCA1_ = 60.3 Hz^2^, σ^2^_C57BL/6 vCA1_/σ^2^_APP/PS1 vCA1_, 95% CI = 0.93–2.42, p = 0.95). Grey areas denote the 95% CI for the null distribution and blue areas denote the areas outside the 95% CI. Orange line denotes variance ratio of 1.0.
(EPS)

S8 Fig Pairwise correlations of excitatory neurons in APP/PS1 mice are decreased in dCA1, but increased in vCA1. (A) Matrices of correlation coefficients for all excitatory neurons in representative recording sessions. (B) In dCA1, mean excitatory neuron correlations were lower in APP/PS1 mice than in C57BL/6 mice (mean ± std: C57BL/6 = 0.048 ± 0.071, APP/PS1 = 0.027 ± 0.049, p < 10^−6^, two-sided Wilcoxon rank-sum test, n_C57BL/6_ = 500 unit pairs from 5 recording sessions, n_APP/PS1_ = 400 unit pairs from 4 recording sessions). (C) In vCA1, mean excitatory neuron correlations were higher in APP/PS1 mice than in C57BL/6 mice (mean ± std: C57BL/6 = 0.010 ± 0.020, APP/PS1 = 0.012 ± 0.018, p < 0.005, two-sided Wilcoxon rank-sum test, n_C57BL/6_ = 600 unit pairs from 6 recording sessions, n_APP/PS1_ = 800 unit pairs from 8 recording sessions). (D) In dCA1, APP/PS1 mice had significantly lower excitatory neuron correlations than C57BL/6 mice when spiking activity was binned at 5ms, 25ms, 50ms, 75ms, and 100ms (for all bin sizes, p < 10^−6^, two-sided Wilcoxon rank-sum test, n_C57BL/6_ = 500 unit pairs from 5 recording sessions, n_APP/PS1_ = 400 unit pairs from 4 recording sessions). Error bars show the standard error of the mean. (E) In vCA1, APP/PS1 mice had significantly higher excitatory neuron correlations than C57BL/6 mice when spiking activity was binned at 5ms, 25ms, 50ms, 75ms, and 100ms (for all bin sizes, p < 0.005, two-sided Wilcoxon rank-sum test, n_C57BL/6_ = 600 unit pairs from 6 recording sessions, n_APP/PS1_ = 800 unit pairs from 8 recording sessions). Error bars show the standard error of the mean. (F) Graph visualizations of 16-unit excitatory neuron populations. Circles denote neurons and lines denote correlations that exceeded a threshold of 0.06. The spatial position of each neuron corresponds to its approximate relative physical location. Scale bars denote 100μm. (G) In dCA1 graphs, the mean degree was smaller in APP/PS1 mice than in C57BL/6 mice (mean ± std: C57BL/6 = 0.65 ± 0.11, APP/PS1 = 0.56 ± 0.61, p < 10^−6^, two-sided Wilcoxon rank-sum test, n_C57BL/6_ = 500 samples from 5 recording sessions, n_APP/PS1_ = 400 samples from 4 recording sessions). (H) In vCA1 graphs, the mean degree was larger in APP/PS1 mice than in C57BL/6 mice (mean ± std: C57BL/6 = 0.49 ± 0.16, APP/PS1 = 0.62 ± 0.14, p < 10^−6^, two-sided Wilcoxon rank-sum test, n_C57BL/6_ = 600 samples from 6 recording sessions, n_APP/PS1_ = 800 samples from 8 recording sessions). (I) In dCA1 graphs, the clustering coefficient was smaller in APP/PS1 mice than in C57BL/6 mice (mean ± std: C57BL/6 = 0.85 ± 0.06, APP/PS1 = 0.80 ± 0.07, p < 10^−6^, two-sided Wilcoxon rank-sum test, n_C57BL/6_ = 500 samples from 5 recording sessions, n_APP/PS1_ = 400 samples from 4 recording sessions). (J) In vCA1 graphs, the clustering coefficient was larger in APP/PS1 mice than in C57BL/6 mice (mean ± std: C57BL/6 = 0.69 ± 0.14, APP/PS1 = 0.79 ± 0.09, p < 10^−6^, two-sided Wilcoxon rank-sum test, n_C57BL/6_ = 600 samples from 6 recording sessions, n_APP/PS1_ = 800 samples from 8 recording sessions).
(EPS)

S9 Fig Differences in entropy between C57BL/6 and APP/PS1 mice are preserved after controlling for firing rate differences. (A) For every 16-unit subsample taken in the C57BL/6 populations in dCA1, a 16-unit subsample with a comparable firing rate (±0.1Hz) was identified from the APP/PS1 dCA1 populations (mean ± std: C57BL/6 = 3.88 ± 1.36Hz, APP/PS1 = 3.89 ± 1.35Hz, p = 0.14, two-sided Wilcoxon sign-rank test, n_C57BL/6_ = 2500 samples from 5 recording sessions, n_APP/PS1_ = 2500 samples from 4 recording sessions). (B) For every 16-unit subsample taken in the C57BL/6 populations in vCA1, a 16-unit subsample with a comparable firing rate (±0.1Hz) was identified from the APP/PS1 vCA1 populations (mean ± std: C57BL/6 = 4.83 ± 1.62Hz, APP/PS1 = 4.83 ± 1.32Hz, p = 0.06, two-sided Wilcoxon sign-rank test, n_C57BL/6_ = 4000 samples from 6 recording sessions, n_APP/PS1_ = 4000 samples from 8 recording sessions). (C) When restricted to dCA1 populations with matched mean firing rates, APP/PS1 mice had significantly decreased entropy relative to C57BL/6 controls (mean ± std: C57BL/6 = 311 ± 74 bits/s, APP/PS1 = 309 ± 69 bits/s, p < 10^−4^, two-sided Wilcoxon sign-rank test, n_C57BL/6_ = 2500 samples from 5 recording sessions, n_APP/PS1_ = 2500 samples from 4 recording sessions). (D) When restricted to vCA1 populations with matched mean firing rates, APP/PS1 mice had significantly increased entropy relative to C57BL/6 controls (mean ± std: C57BL/6 = 344 ± 80 bits/s, APP/PS1 = 358 ± 81 bits/s, p < 10^−6^, two-sided Wilcoxon sign-rank test, n_C57BL/6_ = 4000 samples from 6 recording sessions, n_APP/PS1_ = 4000 samples from 8 recording sessions).
(EPS)

S10 Fig Maximum entropy model per-pattern prediction errors increase for pattern categories with higher numbers of coactive units. (A) In dCA1, the prediction error per pattern in APP/PS1 mice was generally lower than that for C57BL/6 mice across almost all pattern categories. Pattern probabilities were averaged across 2500 subsamples from 5 recording sessions in C57BL/6 mice and across 2000 subsamples from 4 recording sessions in APP/PS1 mice. These data were generated using 16-unit subpopulations. Error bars show the standard error of the mean. (B) In vCA1, the prediction error per pattern in APP/PS1 mice was generally higher than that for C57BL/6 mice across almost all pattern categories. Pattern probabilities were averaged across 3000 subsamples from 6 recording sessions in C57BL/6 mice and across 4000 subsamples from 8 recording sessions in APP/PS1 mice. These data were generated using 16-unit subpopulations. Error bars show the standard error of the mean.
(EPS)

S11 Fig Pairwise correlations during SWR and non-SWR epochs exclusively. (A) Left: During non-SWR epochs, in dCA1, mean correlations were lower in APP/PS1 mice than in C57BL/6 mice (mean ± std: C57BL/6 = 0.14 ± 0.11, APP/PS1 = 0.11 ± 0.12, p < 10^−6^, two-sided Wilcoxon rank-sum test, n_C57BL/6_ = 479 unit pairs from 5 recording sessions, n_APP/PS1_ = 398 unit pairs from 4 recording sessions). Right: During non-SWR epochs, in vCA1, mean correlations were higher in APP/PS1 mice than in C57BL/6 mice (mean ± std: C57BL/6 = 0.09 ± 0.10, APP/PS1 = 0.11 ± 0.11, p < 0.05, two-sided Wilcoxon rank-sum test, n_C57BL/6_ = 591 unit pairs from 6 recording sessions, n_APP/PS1_ = 778 unit pairs from 8 recording sessions). (B) Left: During SWR epochs, in dCA1, mean correlations were lower in APP/PS1 mice than in C57BL/6 mice (mean ± std: C57BL/6 = 0.16 ± 0.14, APP/PS1 = 0.13 ± 0.12, p < 0.005, two-sided Wilcoxon rank-sum test, n_C57BL/6_ = 500 unit pairs from 5 recording sessions, n_APP/PS1_ = 386 unit pairs from 4 recording sessions). Right: During SWR epochs, in vCA1, mean correlations not significantly different in APP/PS1 and C57BL/6 mice (mean ± std: C57BL/6 = 0.11 ± 0.11, APP/PS1 = 0.11 ± 0.11, p = 0.77, two-sided Wilcoxon rank-sum test, n_C57BL/6_ = 589 unit pairs from 6 recording sessions, n_APP/PS1_ = 796 unit pairs from 8 recording sessions).
(EPS)
